# Ubiquitin-specific protease 7 promotes the growth and oncogenic potential of acute myeloid leukemia cells through the deubiquitination and upregulation of LRRK2

**DOI:** 10.1016/j.jbc.2025.110675

**Published:** 2025-09-02

**Authors:** Joon Hyung Park, Kwang Chul Chung

**Affiliations:** Department of Systems Biology, College of Life Science and Biotechnology, Yonsei University, Seoul, South Korea

**Keywords:** LRRK2, USP7, deubiquitinating enzyme, ubiquitination, cancer, AML

## Abstract

Leucine-rich repeat kinase 2 (LRRK2), a large protein with kinase and GTPase activities, regulates various cellular pathways, including autophagy, endocytosis, and mitochondrial dynamics. LRRK2, extensively studied in the context of Parkinson's disease is functionally impaired in other pathological conditions as well, including inflammatory bowel disease, cancer, and cardiovascular diseases. Despite its critical functions, the mechanisms controlling LRRK2 protein stability are not fully understood. Recent studies suggest that the ubiquitin-proteasome system plays a key role in regulating LRRK2 stability. However, the relationship between deubiquitinating enzymes and LRRK2 has not been fully understood. In this study, we identified ubiquitin-specific protease 7 (USP7) as the novel deubiquitinating enzyme that positively regulates LRRK2 by preventing its degradation through ubiquitin-proteasome system. We demonstrated that USP7 directly binds to LRRK2 and promotes its accumulation by deubiquitinating K48-linked polyubiquitin chains. Notably, among various types of cancer, the highest and most significant expression of these two genes was observed in acute myeloid leukemia (AML). We also found that inhibition or knockdown of USP7 suppressed AML cell growth *via* downregulation of LRRK2, and this effect was partially reversed by LRRK2 overexpression. Furthermore, LRRK2 overexpression significantly increased both the colony formation and cell invasion rates in AML cells, compared to the downregulation of USP7. Taken together, our findings identify USP7 as a novel deubiquitinating enzyme of LRRK2 that positively regulates its stability and plays an oncogenic role in AML, with implications for AML cancer progression and potential therapeutic targets.

Leucine-rich repeat kinase 2 (LRRK2) is a large multi-domain protein that possesses both kinase and GTPase activities. LRRK2 has been recognized for its role in multiple cellular processes, including maintaining cellular homeostasis and responding to stress signals. In particular, LRRK2's kinase activity is crucial for the regulation of several signaling pathways by phosphorylating a diverse range of substrates. Among its substrates are members of the Rab GTPase family, synaptojanin, AP2M1, endophilin A1, NADPH oxidase 2, and several transcription factors such as p53 and FOXO1 (1, 2). These interactions regulate processes like autophagy, the organization of the Golgi network, vesicular trafficking, and the immune response, contributing to LRRK2's broad impact on cellular function. Despite the knowledge of LRRK2's extensive roles in cellular signaling, the mechanisms that regulate its stability, particularly through post translational modifications, are still not well understood.

In terms of LRRK2 stability, previous studies have shown that it is subject to regulation by both the ubiquitin-proteasome system (UPS) and the autophagy-lysosomal pathway. Specifically, LRRK2 degradation has been linked to ubiquitin E3 ligases, such as TRIM1 and CHIP, which mediate the UPS degradation of LRRK2 (3, 4, 5). Many ubiquitination targets have been shown to undergo reversible conjugation to ubiquitin by deubiquitinating enzymes (DUBs). The DUBs function to remove ubiquitin molecules from substrates, thereby regulating protein stability, degradation, and many cellular processes. In this context, it can be anticipated that reversible regulation of LRRK2 stability by DUBs may also occur. The DUBs involved in this process consist of seven distinct types, including ubiquitin-specific protease (USP), ubiquitin C-terminal hydrolase, and ovarian tumor domain-containing protease, each with unique characteristics and functions. Despite the crucial roles of LRRK2, little is understood about the DUBs that stabilize the LRRK2 protein.

Although LRRK2 has been extensively studied as a key genetic factor in Parkinson's disease (PD), its involvement in cancer is becoming an area of growing interest. Emerging studies indicate that LRRK2 is implicated in the progression of various cancers, including lung, renal, thyroid, and glioblastoma (6, 7, 8, 9). In particular, LRRK2’s role in regulating immune responses has significant implications for cancer. For example, in pancreatic ductal adenocarcinoma, LRRK2 phosphorylates the programmed cell death ligand 1 (PD-L1), a protein that plays a key role in immune evasion by inhibiting T-cell function. Phosphorylation by LRRK2 prevents the UPS-mediated degradation of PD-L1, thus enhancing its stability and impairing immune responses to the tumor (10, 11). In addition, *LRRK2*-knockdown inhibits NLRP3 inflammasome activation while promoting the NF-κB signaling pathway, further contributing to cancer progression by modulating inflammatory responses and cellular survival mechanisms. These findings suggest that LRRK2 not only contributes to PD but also plays a key role in cancer biology, particularly through immune modulation. Like LRRK2, USPs are deeply involved in the development of many cancers.

Among the various USPs, USP7 regulates a broad range of cellular pathways, including the cell cycle, DNA replication, and apoptosis (12, 13). USP7 is also associated with cancer development and plays both oncogenic and tumor suppressor roles depending on the cancer type and substrate. In some cancers, such as cervical cancer, elevated USP7 expression is associated with enhanced tumor progression. This occurs *via* the upregulation of EZH2, which subsequently inhibits the expression of TIMP2, promoting the activation of PD-L1 and enabling immune escape mechanisms in the tumor microenvironment (14, 15). Furthermore, USP7 regulates the NLRP3 inflammasome, with studies showing that inhibition of USP7 increases the ubiquitination of NLRP3 and suppresses inflammasome activity, pointing to its critical role in regulating inflammation and immune responses (14).

Interestingly, both LRRK2 and USP7 share common substrates, including critical transcription factors such as p53, FOXO1, and FOXO4, which are essential for regulating cell survival, proliferation, and stress responses (16, 17, 18, 19, 20). The interaction between these two proteins may significantly influence the transcriptional activities of these factors, as well as immune responses. Given that both LRRK2 and USP7 are involved in modulating critical pathways associated with cancer progression, it is plausible that their functional relationship may be of particular importance in tumorigenesis.

Based on these findings, in the present study we explored the potential linkage between USP7 and LRRK2 with a particular focus on how USP7 may positively modulate LRRK2 stability. Furthermore, we examined how alterations in this interaction could contribute to cancer progression in specific malignancies. Our results demonstrated that USP7 acts as a novel DUB for LRRK2, preventing UPS-mediated LRRK2 degradation and causing its accumulation. Finally, we have shown that the increase of LRRK2 *via* USP7 may play an oncogenic role in acute myeloid leukemia (AML), highlighting the importance of the USP7-LRRK2 interaction in cancer biology.

## Results

### The expression of both LRRK2 and USP7 gene was elevated in the AML cells compared to other cancer cell lines

**Figure 1:**
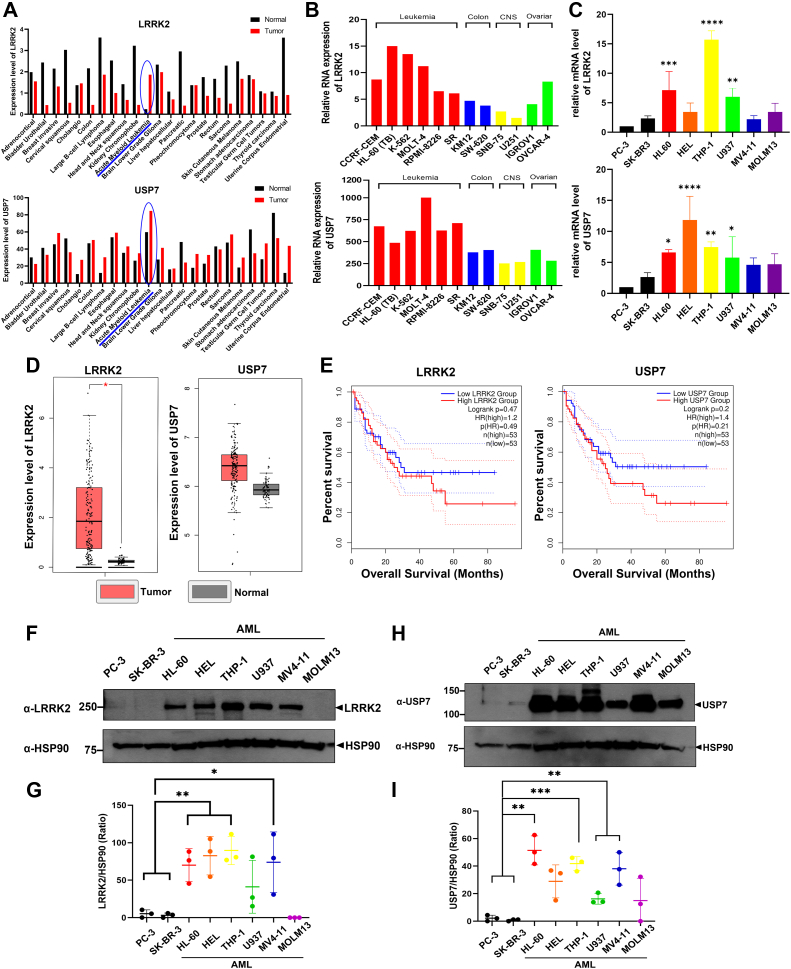
***LRRK2* and *USP7* gene expression was increased in AML.***A*, analysis of the TCGA database was conducted using GEPIA2. The expression levels of USP7 and LRRK2 genes in cancer tissues, relative to those in various normal tissues, are shown. The values on the *Y*-axis represent the median expression of the Log2-normalized transcription frequency of the target gene in both cancer and normal tissues. *B*, microarray-based expression data for LRRK2 (Exp. ID: 113136; https://dtp.cancer.gov/mtweb/targetinfo?moltid=GC208403&moltnbr=113136) and USP7 (Exp. ID: 222104; https://dtp.cancer.gov/mtweb/targetinfo?moltid=GC182522&moltnbr=222104) were obtained from the U.S. National Cancer Institute (NCI) database (http://dtp.nci.nih.gov/mtweb/targetdata). The dataset includes gene expression profiles across various human cancer cell lines, including leukemia, colon, CNS, and ovarian cancers. *C*, quantitative real-time PCR (qRT-PCR) was performed to assess the mRNA expression levels of LRRK2 and USP7 in various AML cell lines, using non-hematologic cancer cell lines PC-3 and SK-BR-3 as references. Data are presented as the mean ± SD from three independent experiments (∗∗∗∗*p* ≤ 0.0001; ∗∗∗*p* ≤ 0.001 ∗∗*p* ≤ 0.01; ∗*p* ≤ 0.05). *D*, the median expression levels of *LRRK2* and *USP7* genes in AML cancer tissues, compared to normal blood cells. GEPIA2 analysis, based on TCGA data, shows that the expression levels of LRRK2 and USP7 are increased in AML tissues (n = 173) compared to normal tissues (n = 70) (∗*p* ≤ 0.05). *E*, the overall survival rate of AML patients was assessed through analysis of TCGA data from GEPIA2, with patients (n = 53) grouped based on high expression levels of LRRK2 or USP7 (*red*) *versus* low expression levels of LRRK2 or USP7 (*blue*). *F*, immunoblotting was performed using an anti-LRRK2 antibody on various cell lines (PC-3, SK-BR-3) and AML cell lines (HL-60, HEL, THP-1, U937, and MV4-11, MOLM13) to assess LRRK2 expression levels. *G*, the expression levels of LRRK2 were quantified, and the resulting data were normalized to HSP90. Data are presented as the mean ± SD obtained from three independent experiments (∗∗*p* ≤ 0.01; ∗*p* ≤ 0.05). *H*, immunoblotting was performed using an anti-USP7 antibody on various cell lines (PC-3, SK-BR-3) and six AML cell lines to assess LRRK2 expression levels. *I*, the expression levels of USP7 were quantified, and the resulting data were normalized to HSP90. Data are presented as the mean ± SD obtained from three independent experiments (∗∗∗*p* ≤ 0.001; ∗∗*p* ≤ 0.01). NCI = National Cancer Institute; TCGA = The Cancer Genome Atlas; GEPIA2 = Gene Expression Profiling Interactive Analysis application 2. HSP90 served as a loading control. AML, acute myeloid leukemia; LRRK2, leucine-rich repeat kinase 2; USP7, ubiquitin-specific protease 7.

Building on previous studies that have highlighted the involvement of *LRRK2* and *USP7* in oncogenesis across various cancers, including lung, thyroid, and renal cancers (6, 7, 8, 9, 16), we hypothesized that the expression of both USP7 and LRRK2 may be commonly associated with the development of specific cancer types. To test this hypothesis, we first examined the relationship between the expression levels of LRRK2 and USP7 across different cancer types. For this analysis, we used the Gene Expression Profiling Interactive Analysis 2 (GEPIA2) and identify the cancers with the highest expression of both LRRK2 and USP7. GEPIA2 analysis revealed that LRRK2 and USP7 expression levels were markedly elevated in AML compared to other cancer types (Fig. 1*A*).

Complementing the GEPIA2 results, analysis of microarray data from the U.S. National Cancer Institute (NCI) database further demonstrated that *LRRK2* mRNA levels were markedly elevated across various leukemia cell lines relative to colon, central nervous system, and ovarian cancer cell lines. A similar pattern of elevated expression was observed for *USP7* in the leukemia cell lines (Fig. 1*B*). Thus, while GEPIA2 analysis pointed to transcriptional upregulation of *LRRK2* and *USP7* specifically in AML, the NCI database revealed broader overexpression across multiple leukemia subtypes, including acute lymphoblastic leukemia, AML, chronic lymphocytic leukemia (CLL), and chronic myeloid leukemia. To validate these observations, we performed quantitative real-time PCR (qRT-PCR) to measure *LRRK2* and *USP7* mRNA expression in a panel of AML cell lines, using PC-3 (prostate cancer) and SK-BR-3 (breast cancer) as non-hematologic controls. Consistent with the database results, the majority of AML cell lines exhibited significantly higher mRNA levels of both *LRRK2* and *USP7* compared to the controls (Fig. 1*C*). Further analysis of data from The Cancer Genome Atlas confirmed that the expression levels of both LRRK2 and USP7 were significantly higher in AML compared to normal blood cells (Fig. 1*D*).

Next, we hypothesized that the increased expression of LRRK2 and USP7 in AML might impact the overall survival of AML patients, prompting further investigation. Analysis of The Cancer Genome Atlas data through GEPIA2 revealed that patients with high expression levels of both LRRK2 and USP7 had reduced overall survival compared to those with low expression levels (Fig. 1*E*). Based on these findings, we hypothesized that LRRK2 and USP7 may play critical roles in AML progression and patient prognosis. To further explore this, we examined whether intracellular LRRK2 expression is elevated in various AML cell lines. Immunoblotting of cancer cell lysates, including those from human prostate cancer (PC-3), breast cancer (SK-BR-3), and AML cell lines (HL-60, HEL, THP-1, U937, MV4-11, and MOLM13) using an anti-LRRK2 antibody showed that LRRK2 expression was particularly elevated in all AML cell lines except MOLM13 (Fig. 1, *F* and *G*). In addition, intracellular USP7 expression was assessed across the same cell lines and was found to be markedly elevated in AML cell lines, including HL-60, HEL, THP-1, U937, MV4-11, and MOLM13 (Fig. 1, *H* and *I*). Notably, the expression levels of both LRRK2 and USP7 were significantly higher in the THP-1 cell line. These results suggest that LRRK2 and USP7 are expressed at higher levels in AML cell lines compared to other cancer cell lines, implying that they may contribute to the progression and development of AML.

### USP7 interacts with LRRK2 in mammalian cells

**Figure 2:**
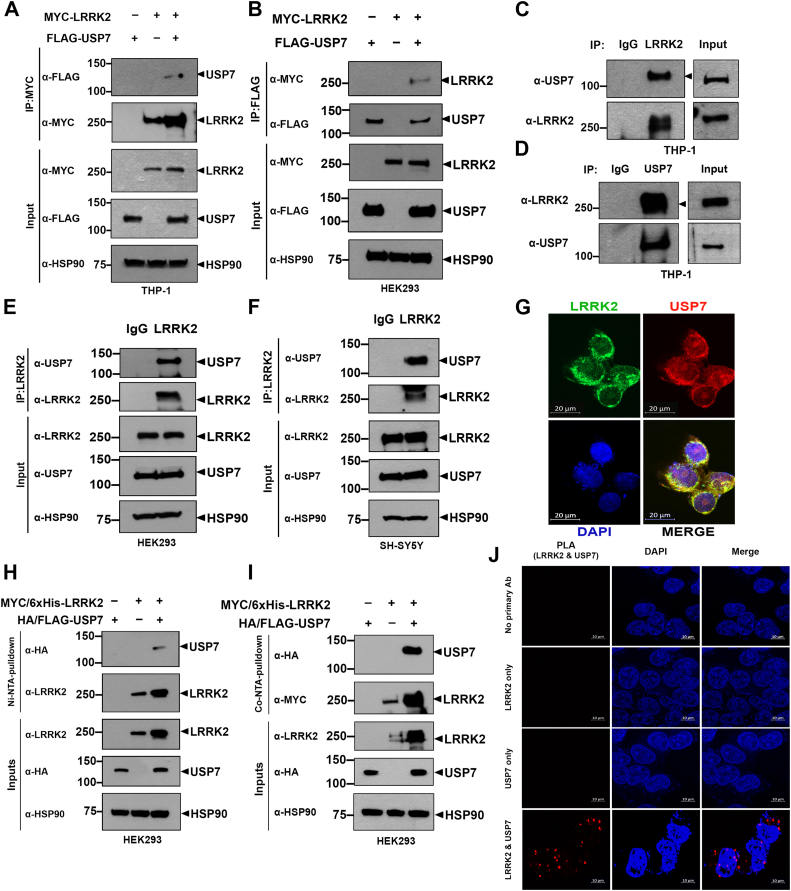
**USP7 interacts with LRRK2 in mammalian cells.***A* and *B*, THP-1 (*A*) and HEK293 cells (*B*) were transfected with plasmids encoding Myc-LRRK2 alone or together with Flag-USP7 for 24 h. Cell lysates were subjected to immunoprecipitation using anti-Myc or anti-Flag antibodies, followed by immunoblotting using the indicated antibodies. *C* and *D*, where indicated, whole cell lysates from THP-1 cells were subjected to immunoprecipitation using anti-LRRK2 (*C*), anti-USP7 (*D*), or immunoglobulin G (IgG) as a control, followed by immunoblotting with the indicated antibodies. *E* and *F*, HEK293 (*E*) or SH-SY5Y cells (*F*) were immunoprecipitated using anti-LRRK2 antibody or IgG as a control, and immunoblotting with the indicated antibodies was performed. *G*, after treating the dish with poly-D-lysine for 2 h, HEK293 cells were plated and cultured for 24 h. Following cell fixation, endogenous LRRK2 and USP7 were stained with their antibodies. Representative confocal images of endogenous immunostaining are shown, with LRRK2 (*green*) and USP7 (*red*) stained using their corresponding antibodies. The scale bars represent 20 μm. DAPI (*blue*) was used for nuclear staining. *H* and *I*, after HEK293 cells were transfected for 24 h with plasmids encoding LRRK2-Myc/6xHis or HA/Flag-USP7 alone or together, cell lysates were incubated with Ni^2+^-NTA beads for 1 h (*H*), followed by elution with imidazole. Immunoblotting was then performed using the indicated antibodies. *I*, cell lysates were incubated with Co^2+^-NTA beads for pull-down, and immunoblotting was carried out with the indicated antibodies. HSP90 served as a loading control. *J*, PLAs were performed using primary antibodies against LRRK2 and USP7. Representative PLA images (*red*) depicting the interaction between endogenous LRRK2 and USP7 are shown. DAPI (*blue*) was used for nuclear staining. The scale bars represent 10 μm. DAPI, 4′, 6-diamidino-2-phenylindole; HA, hemagglutinin; HEK293, human embryonic kidney 293; IgG, immunoglobulin G; LRRK2, leucine-rich repeat kinase 2; PLA, proximity ligation assay; USP7, ubiquitin-specific protease 7.

We next investigated whether USP7 interacts with LRRK2 in mammalian cells. In THP-1 cells transfected with plasmids encoding Flag-tagged USP7, Myc-tagged LRRK2, or both, coimmunoprecipitation (co-IP) using an anti-Myc antibody followed by immunoblotting with an anti-Flag antibody confirmed that ectopically expressed USP7 binds to LRRK2 (Fig. 2*A*). A similar co-IP experiment in human embryonic kidney 293 (HEK293) cells yielded consistent results (Fig. 2*B*). To test endogenous interaction, co-IP was performed in THP-1 cells using either anti-USP7 or anti-LRRK2 antibodies, with preimmune immunoglobulin G (IgG) as a negative control. Immunoblot analysis confirmed that endogenous USP7 and LRRK2 form a complex in THP-1 cells (Fig. 2, *C* and *D*). This interaction was further validated in additional mammalian cell lines—HEK293 and SH-SY5Y—where co-IP of endogenous proteins showed that LRRK2 and USP7 also associate in these contexts (Fig. 2, *E* and *F*). Immunofluorescence staining in HEK293 cells revealed colocalization of USP7 and LRRK2 in the cytosol (Fig. 2*G*), further supporting their interaction.

To provide additional biochemical evidence, we performed Ni-NTA and Co-NTA pull-down assays using lysates from HEK293 cells co-transfected with HA/Flag-tagged USP7 and Myc-6xHis-tagged LRRK2 constructs. His-tagged LRRK2 was isolated *via* metal-affinity chromatography using nickel or cobalt resin, which selectively binds histidine residues. Immunoblotting of the eluted complexes demonstrated that USP7 copurified with His-tagged LRRK2, further supporting a physical association between the two proteins (Fig. 2, *H* and *I*). To complement these findings and assess the subcellular proximity of LRRK2 and USP7 at high resolution, we performed a proximity ligation assay (PLA), which detects proteins that are within ∼40 nm of each other, thus indicating direct or near-direct interactions. A strong PLA signal was observed between LRRK2 and USP7, consistent with their close spatial proximity and further validating the interaction at the endogenous level (Fig. 2*J*).

Together, these results provide multiple lines of evidence that USP7 physically interacts with LRRK2 in mammalian cells, including THP-1 AML cells.

### LRRK2 binds to the TRAF and UBL-3, -4, and -5 domains of USP7

Next, we examined which regions of USP7 bind to full-length LRRK2. USP7 consists of three domains: The N-terminal TRAF domain, the catalytic domain, and the C-terminal five UB-like (UBL) subdomains (U1-U5). To investigate this interaction, we used four serial USP7-deletion mutants (D1–D4). To determine the specific USP7 domains responsible for binding LRRK2, we prepared HEK293 cell lysates expressing WT LRRK2 or full-length USP7, along with USP7 deletion mutants: the TRAF domain (1–208; D1), the catalytic domain (206–560; D2), the first two UBL subdomains (560–776 a.a., U1+U2; D3), and the last three UBL subdomains (776–1102 a.a., U3-U5; D4) (Fig. S1*A*). These lysates were subjected to co-IP assays. Immunoblotting analysis revealed that LRRK2 binds not only to full-length USP7 but also to the USP7-D1 and -D4 fragments, which correspond to the 1 to 208 and 776 to 1102 regions (Fig. S1*B*). However, no binding was observed with USP7-D2 and D3 fragments. These results suggest that LRRK2 interacts with USP7 through its TRAF domain and the C-terminal three UBL subdomains.

#### USP7 upregulates the expression level of LRRK2

**Figure 3:**
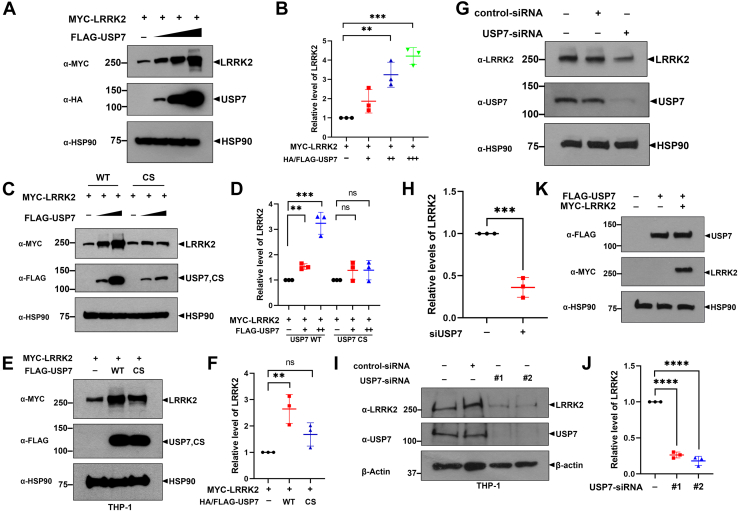
**USP7 upregulates the expression level of LRRK2.***A*, HEK293 cells were transfected for 24 h with plasmids encoding Myc-LRRK2 alone or together with increasing dose of Flag-USP7. Cell lysates were then subjected to immunoblotting using the indicated antibodies. *B*, relative LRRK2 levels were quantified, and data are presented as the mean ± SD of three independent experiments (∗∗∗*p* ≤ 0.001; ∗∗*p* ≤ 0.01). *C*, HEK293 cells were transfected for 24 h with a plasmid encoding Myc-LRRK2 alone or in combination with increasing doses of Flag-USP7-WT or Flag-USP7-C223S (USP7-CS). Cell lysates were then subjected to immunoblotting using the indicated antibodies. *D*, relative LRRK2 levels were quantified, and data are presented as the mean ± SD of three independent experiments (∗∗∗*p* ≤ 0.001; ∗∗*p* ≤ 0.01; NS, not significant). *E*, THP-1 cells were transfected for 24 h with a plasmid encoding Myc-LRRK2 alone or together with Flag-USP7-WT or Flag-USP7-CS. Subsequently, immunoblotting was performed using the indicated antibodies. *F*, relative LRRK2 levels were quantified, and data are presented as the mean ± SD of three independent experiments (∗∗*p* ≤ 0.01; NS, not significant). *G*, HEK293 cells were transfected with control-siRNA or *USP7*-siRNA for 48 h. Cell lysates were then subjected to immunoblotting using anti-LRRK2 or anti-USP7 antibodies. *H*, the relative expression levels of LRRK2 following *USP7*-knockdown were measured, and the results are presented as the mean ± SD of three independent experiments. (∗∗∗*p* ≤ 0.001). *I*, THP-1 cells were subjected to siRNA-mediated *USP7*-knockdown for 48 h, followed by immunoblotting with anti-LRRK2 or anti-USP7 antibodies. *J*, the relative expression levels of LRRK2 following *USP7*-knockdown were measured, and the results are presented as the mean ± SD of three independent experiments (∗∗∗∗*p* ≤ 0.0001). HSP90 and β-actin were served as a loading control. *K*, HEK293 cells were transfected for 24 h with plasmids encoding Flag-USP7 alone or together with Myc-LRRK2. Cell lysates were then subjected to immunoblotting using the indicated antibodies. HSP90 served as a loading control. HEK293, human embryonic kidney 293; LRRK2, leucine-rich repeat kinase 2; USP7, ubiquitin-specific protease 7.

To further investigate the relationship between USP7 and LRRK2, we examined whether USP7 regulates LRRK2 expression or *vice versa*. First, Phos-tag gel-based immunoblotting showed that the phosphorylation status of USP7 remained unchanged in the presence of LRRK2, suggesting that LRRK2 does not modulate USP7 phosphorylation under the tested conditions (Fig. S2*A*). Consistently, in both THP-1 and HEK293 cells transfected with plasmids encoding Myc-LRRK2 or Flag-USP7, either alone or in combination, USP7 banding patterns remained unaffected regardless of LRRK2 expression, confirming that LRRK2 has no detectable effect on USP7 stability or modification (Figs. 2, *A*, *B* and 3*K*). In contrast, the protein levels of exogenously expressed LRRK2 increased in the presence of USP7 in a dose-dependent manner (Figs. 2, *A*, *B* and 3, *A*, *B*).

To assess whether this regulation is linked to proteasomal degradation, we treated cells with the proteasome inhibitor MG132. Endogenous LRRK2 levels increased in a dose-dependent manner following MG132 treatment, whereas endogenous USP7 levels remained largely unaffected (Fig. S3, *A*–*D*), indicating that LRRK2, but not USP7, is subject to proteasome-mediated degradation.

To determine whether the catalytic activity of USP7 is required for LRRK2 upregulation, HEK293 cells were transfected with Myc-LRRK2 along with increasing amounts of either wild-type USP7 (USP7-WT) or a catalytically inactive mutant (USP7-C223S, hereafter USP7-CS). Only USP7-WT, not USP7-CS, induced a dose-dependent increase in LRRK2 levels (Fig. 3, *C* and *D*), a result that was similarly reproduced in AML cell lines (Fig. 3, *E* and *F*). We further assessed the impact of USP7 depletion on LRRK2 expression. *USP7*-knockdown *via* siRNA in HEK293 cells led to over 50% reduction in endogenous LRRK2 levels (Fig. 3, *G* and *H*), and similar knockdown in THP-1 cells resulted in an even greater decrease of approximately 70 to 80% (Fig. 3, *I* and *J*). Conversely, LRRK2 overexpression did not affect USP7 protein levels (Fig. 3*K*), supporting the notion that the regulatory relationship is unidirectional.

Together, these findings demonstrate that USP7 positively regulates LRRK2 protein expression in mammalian cells through a mechanism dependent on its deubiquitinating activity.

#### USP7 increases the protein stability of LRRK2

**Figure 4:**
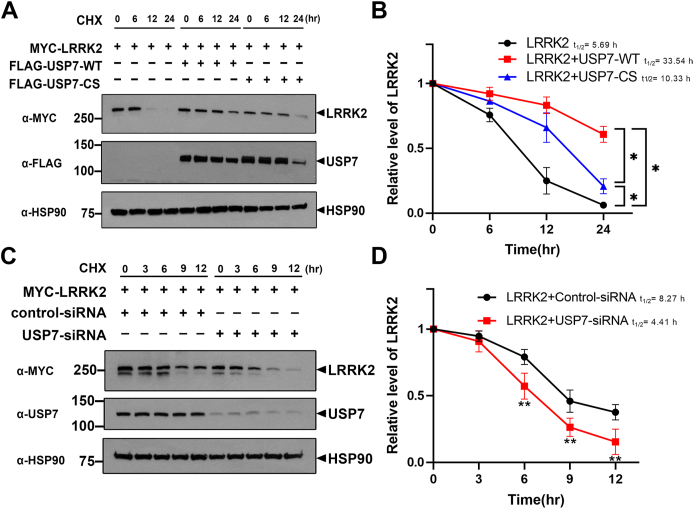
**USP7 increases the protein stability of LRRK2.***A*, HEK293 cells were transfected for 24 h with plasmid encoding Myc-LRRK2 alone or together with FLAG-USP7-WT or FLAG-USP7-CS. Cells were then treated with 100 μg/ml of cycloheximide for the indicated times, and the cell lysates were subjected to immunoblotting using the specified antibodies. *B*, relative LRRK2 levels were quantified, and the results are presented as the mean ± SD from three independent experiments (∗*p* ≤ 0.05). *C*, HEK293 cells were transfected for 48 h with control-siRNA or *USP7*-siRNA. Cells were then treated with 100 μg/ml of cycloheximide for indicated times, and the cell lysates were subjected to immunoblotting using the specified antibodies. *D*, relative LRRK2 levels were quantified and the results are presented as the mean ± SD from three independent experiments (∗∗*p* ≤ 0.01). HSP90 served as a loading control. HEK293, human embryonic kidney 293; LRRK2, leucine-rich repeat kinase 2; USP7, ubiquitin-specific protease 7.

To further investigate the effect of USP7 on LRRK2 protein stability, we compared the half-life of LRRK2 in the presence or absence of USP7. HEK293 cells were transfected with plasmids encoding Myc-tagged LRRK2 alone, or cotransfected with either wild-type USP7 (USP7-WT) or its catalytically inactive mutant (USP7-CS). Following transfection, cells were treated with cycloheximide for the indicated time points to block new protein synthesis. Immunoblot analysis revealed that co-expression of USP7-WT significantly increased the half-life of LRRK2 compared to LRRK2 alone, indicating that USP7 stabilizes LRRK2 by inhibiting its degradation (Fig. 4, *A* and *B*). In contrast, co-expression with the USP7-CS mutant had a much weaker effect. As shown in the graph (Fig. 4*B*), the half-life of LRRK2, estimated using an exponential decay model, was approximately 5.7 h when expressed alone. Co-expression with USP7-WT extended the half-life to 33.5 h, whereas co-expression with USP7-CS led to a moderate increase to approximately 10.3 h, supporting the idea that USP7’s catalytic activity is necessary for LRRK2 stabilization. Conversely, knockdown of USP7 using siRNA in HEK293 cells significantly reduced the half-life of endogenous LRRK2 compared to cells treated with control siRNA (Fig. 4, *C* and *D*). Quantitative analysis showed that LRRK2 half-life decreased from 8.3 h (control siRNA) to 4.4 h following USP7 depletion (Fig. 4*D*).

These results collectively indicate that USP7 promotes the protein stability of LRRK2 in a dose-dependent manner on its deubiquitinating activity, most likely by protecting LRRK2 from proteasomal degradation.

#### USP7 reduces K48-linked ubiquitination levels of LRRK2

**Figure 5:**
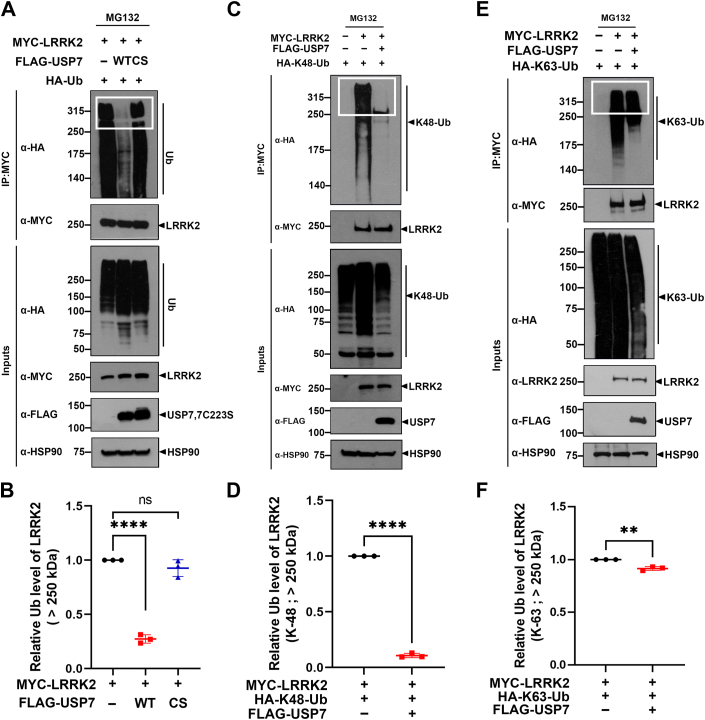
**USP7 reduces K48-linked ubiquitination levels of LRRK2.***A*, HEK293 cells were transfected for 24 h with plasmid encoding Myc-LRRK2 alone or together with either Flag-USP7-WT or Flag-USP7-CS. Following DNA transfection, cells were treated with 20 μM MG132 for an additional 4 h. co-IP was then performed using anti-Myc antibody, and the samples were subsequently immunoblotted with the indicated antibody. *B*, relative Ub level of LRRK2 were quantified, and the data are presented as the mean ± SD of three independent experiments (∗∗*p* ≤ 0.01; NS, not significant). *C*, where indicated, HEK293 cells were transfected for 24 h with plasmids encoding Myc-LRRK2, K48-Ub in which all six lysine residues of ubiquitin are mutated to arginine except K48, or Flag-USP7 WT alone or in combination. After DNA transfection, the cells were treated with 20 μM MG132 for 4 h, followed by immunoprecipitation using an anti-Myc antibody and subsequent immunoblotting with the indicated antibodies. *D*, relative K48-Ub level of LRRK2 were quantified, and the data are presented as the mean ± SD of three independent experiments (∗∗∗∗*p* ≤ 0.0001). *E*, where indicated, HEK293 cells were transfected for 24 h with plasmids encoding Myc-LRRK2, K63-Ub in which all six lysine residues of ubiquitin are mutated to arginine except K63, or Flag-USP7 WT alone or in combination. After DNA transfection, cells were treated with 20 μM MG132 for 4 h, followed by immunoprecipitation using anti-Myc antibody and subsequent immunoblotting with the indicated antibodies. *F*, relative K63-Ub level of LRRK2 were quantified, and the data are presented as the mean ± SD of three independent experiments (∗∗∗*p* ≤ 0.001). HSP90 served as a loading control. Regions corresponding to ubiquitinated LRRK2 species above 250 kDa were quantified and are indicated by *white boxes*. co-IP, coimmunoprecipitation; HEK293, human embryonic kidney 293; LRRK2, leucine-rich repeat kinase 2; USP7, ubiquitin-specific protease 7.

Building on previous findings and our current data showing that LRRK2 is a target of polyubiquitination and that the increase in LRRK2 levels depends on the catalytic activity of USP7, we next examined whether USP7 directly deubiquitinates LRRK2. Ubiquitin (Ub) is covalently attached to substrates through its methionine (M1) residue or one of seven lysine residues (K6, K11, K27, K29, K33, K48, and K63), leading to proteasome-mediated degradation or regulation of the substrate’s biochemical and functional properties. Among these residues, K48-linked and K63-linked ubiquitination are the most common; K48-linked ubiquitination targets proteins for proteasome degradation, while K63-linked ubiquitination induces biochemical or functional changes in the target protein, altering its role in numerous intracellular processes without necessarily leading to degradation (21). To investigate whether USP7 affects LRRK2 ubiquitination and its influence on K48- and K63-linked ubiquitination, we transfected HEK293 cells for 24 h with plasmids encoding HA-Ub and Myc-LRRK2 alone or in combination with Flag-USP7-WT or the Flag-USP7-CS mutant. We then performed co-IP using an anti-Myc antibody. Immunoblot analysis of anti-Myc precipitates with anti-HA antibody revealed that USP7-WT significantly reduced the polyubiquitination level of LRRK2 by approximately 65% (Fig. 5, *A* and *B*). However, this effect was not observed with the USP7-CS mutant (Fig. 5, *A* and *B*). To more directly assess the levels of ubiquitin conjugated to LRRK2 above 250 kDa—and to minimize potential nonspecific effects caused by the migration of smaller proteins or indirect associations within protein complexes—we performed a sequential Ni^2+^-NTA pull-down followed by co-immunoprecipitation. Specifically, cell lysates were first subjected to Ni^2+^-NTA affinity purification to isolate His-tagged ubiquitinated proteins, then further enriched for LRRK2 by immunoprecipitation with an anti-LRRK2 antibody. The resulting eluates were analyzed by immunoblotting. Consistent with the results shown in Figure 5*A*, immunoblotting with anti-HA antibodies revealed that USP7-WT reduced LRRK2 ubiquitination by approximately 80%, whereas the USP7-CS mutant had no such effect (Fig. S4, *A* and *B*).

Next, we investigated which lysine linkages in the polyubiquitin chains of LRRK2 are modulated by USP7. HEK293 cells were transfected with Myc-tagged LRRK2 alone, or co-transfected with HA-tagged ubiquitin constructs containing lysine-to-arginine mutations at all positions except K48 or K63 (*i.e.*, K48 only or K63 only Ub constructs), in the presence or absence of Flag-tagged USP7. After cell lysis, co-IP was performed using an anti-Myc antibody, followed by immunoblotting with an anti-HA antibody. This analysis revealed that USP7 markedly reduced K48-linked polyubiquitination of LRRK2 by approximately 90% (Fig. 5, *C* and *D*).

However, the effect of USP7 on K63-linked ubiquitin was much less pronounced, reducing it by less than 12% (Fig. 5, *E* and *F*). Taken together, these results suggest that USP7, as a novel DUB of LRRK2, primarily catalyzes the deubiquitination of K48-linked ubiquitin chains. This is consistent with our earlier finding that USP7 prevents LRRK2 degradation *via* the UPS.

#### Inhibition of USP7 suppresses AML cell growth, and this effect is attenuated by LRRK2

**Figure 6:**
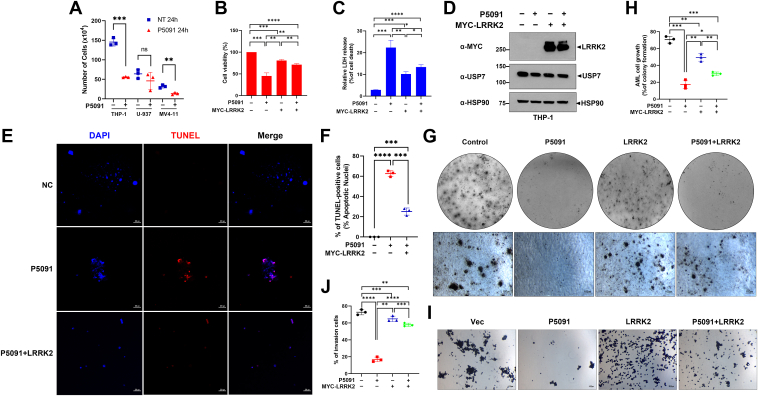
**Inhibition of USP7 suppresses AML cell growth, and it is attenuated by LRRK2.***A*, where indicated, several AML cell lines, including THP-1, U937, and MV4-11 cells, were treated with 20 μM of P5091 for 24 h. Cells were then stained with trypan *blue*, and the number of live cells was quantified. The results are presented as the mean ± SD from three independent experiments (∗∗∗*p* ≤ 0.001; ∗∗*p* ≤ 0.01; NS, not significant). *B*-*H*, after THP-1 cells were mock-transfected or transfected for 24 h with Myc-LRRK2, cells were *left* untreated or treated with 20 μM of P5091 for additional 24 h. *B*, cell viability was measured after 2 h of incubation with Cell Counting Kit-8 (CCK-8) reagent (∗∗∗*p* ≤ 0.001; ∗∗*p* ≤ 0.01). *C*, LDH assay was performed to assess cell death (∗∗∗*p* ≤ 0.001; ∗∗*p* ≤ 0.01; ∗*p* ≤ 0.05). *D*, THP-1 cells were either mock-transfected or transfected for 24 h with a plasmid encoding Myc-LRRK2, followed by treatment with P5091 for additional 24 h. Cell lysates were then subjected to immunoblotting with the indicated antibodies. HSP90 served as a loading control. *E*, after cell fixation with 2% paraformaldehyde and permeabilization, TUNEL reaction was performed with enzyme buffer containing TMR *red*. Fluorescence microscopy was then used to detect TMR red signals from the cells (*red*). DAPI (*blue*) was used for nuclear staining. *F*, the proportion of cells positive for TMR *red* signal was calculated. The results are presented as the mean ± SD from three independent experiments (∗∗∗∗*p* ≤ 0.0001; ∗∗∗*p* ≤ 0.001). *G*, the results of the colony formation assay are shown. After DNA transfection followed by treatment of P5091, cells were then cultured for 21 days in an agarose: media (1:1) mixture. Afterward, colonies were stained with 0.01% *crystal violet* and observed under a microscope. *H*, the colony formation ratio relative to the total area was quantified and expressed as a percentage. The results are presented as the mean ± SD from three independent experiments (∗∗*p* ≤ 0.01). *I*, cell invasion assay results are shown. After 24 h of incubation in the inner chamber of a trans-well, THP-1 cells that invaded into the lower chamber were stained with 0.05% *crystal violet* and observed under a microscope. *J*, the percentage of cells that invaded from the inner chamber of the trans-well to the bottom chamber was quantified. The results are presented as the mean ± SD from three independent experiments (∗∗∗∗*p* ≤ 0.0001; ∗∗∗*p* ≤ 0.001; ∗*p* ≤ 0.05). AML, acute myeloid leukemia; DAPI, 4′, 6-diamidino-2-phenylindole; LRRK2, leucine-rich repeat kinase 2; USP7, ubiquitin-specific protease 7.

We then examined whether USP7’s role in regulating LRRK2 affects the growth of AML cells. To explore this, we used P5091, a specific chemical inhibitor of USP7 (22). Among AML cell lines, we selected three AML cell lines (THP-1, U937, and MV4-11), all of which show high expression of LRRK2 and USP7. After treating the cells with P5091 for 24 h, we measured cell numbers at each time point and compared them with those from the untreated control group. As shown in Figure 6*A*, two out of three AML cell lines showed significantly reduced cell counts in response to P5091 treatment, with THP-1 cells exhibiting the most significant reduction (Fig. 6*A*).

Next, we investigated whether the reduced growth of AML cells due to USP7 inhibition was influenced by LRRK2 expression. THP-1 cells were either mock-transfected or transfected with a plasmid encoding Myc-LRRK2 for 24 h. Cells were then treated with P5091 for an additional 24 h. Cell viability was assessed using a Cell Counting Kit-8 (CCK-8) assay, showing a significant reduction in cell numbers upon USP7 inhibition. Notably, cells transiently transfected with Myc-LRRK2 displayed partial recovery in cell count (Fig. 6*B*).

We then assessed the effect of USP7 inhibition or/and LRRK2 overexpression on AML cell death. After THP-1 cells were mock-transfected or transfected with a plasmid encoding Myc-LRRK2 for 24 h, they were either left untreated or treated with P5091 for another 24 h. Cell death was measured using a lactate dehydrogenase (LDH) assay, revealing a significant increase in cell death upon USP7 inhibition. However, LRRK2 overexpression partially reduced the AML cell death caused by USP7 inhibition (Fig. 6*C*). To evaluate the effects of P5091 on both AML cell growth and the expression levels of LRRK2 and USP7, we performed immunoblot analysis following 24 h of treatment. P5091 treatment led to a moderate reduction in LRRK2 protein levels, whereas USP7 protein levels remained largely unchanged. These results indicate that P5091 does not affect USP7 expression at the protein level, but rather exerts its effects by inhibiting USP7 enzymatic activity (Fig. 6*D*).

Furthermore, to determine whether the increased AML cell death due to USP7 inhibition was driven by apoptosis, we performed a TUNEL assay. This assay detects DNA fragmentation, which occurs specifically during apoptosis, but not other forms of cell death, such as necrosis. After transfecting THP-1 cells with either Myc-LRRK2 or mock-transfection for 24 h, cells were treated with P5091 for an additional 24 h. Following fixation, permeabilization, and TMR red labeling, fluorescence microscopy revealed that USP7 inhibition led to nearly a 60% increase in apoptotic AML cell death (Fig. 6, *E* and *F*). Overexpression of LRRK2 reduced apoptosis by approximately 40%, compared to cells treated with P5091 alone (Fig. 6, *E* and *F*).

We further explored the effects of USP7 inhibition or/and LRRK2 overexpression on AML cell growth using a colony formation assay. After transfecting THP-1 cells with Myc-LRRK2 for 24 h, cells were treated with the USP7 inhibitor for an additional 24 h. The cells were cultured for 21 days, and colonies were stained with 0.01% crystal violet. Microscopic observation showed a significant reduction in colony formation in the USP7 inhibitor-treated group (Fig. 6, *G* and *H*). In contrast, combined USP7 inhibition and Myc-LRRK2 transfection led to an increase in colony formation (Fig. 6, *G* and *H*).

Next, we investigated the effect of USP7 inhibition and/or LRRK2 expression on AML cell invasion using a trans-well assay. THP-1 cells treated with the USP7 inhibitor displayed reduced invasion, but this was partially restored in cells transfected with Myc-LRRK2 followed by treatment with the USP7 inhibitor (Fig. 6, *I* and *J*).

To further validate the findings shown in Figure 6, we repeated key experiments using HBX19818, another selective USP7 inhibitor (23), as an alternative to P5091. The results, shown in Fig. S5, closely mirrored those obtained with P5091, reinforcing the conclusion that the observed effects are attributable to USP7 inhibition rather than off-target activity (Fig. S5, *A*–*H*).

Taken together, these results suggest that inhibition of USP7 reduces AML cell growth and increases apoptotic cell death. This effect was further supported by the reduction in colony formation and cell invasion, which could be partially rescued by LRRK2 overexpression.

#### Inhibition of AML cell growth and its oncogenic properties by USP7-knockdown is partially restored by LRRK2 overexpression

**Figure 7:**
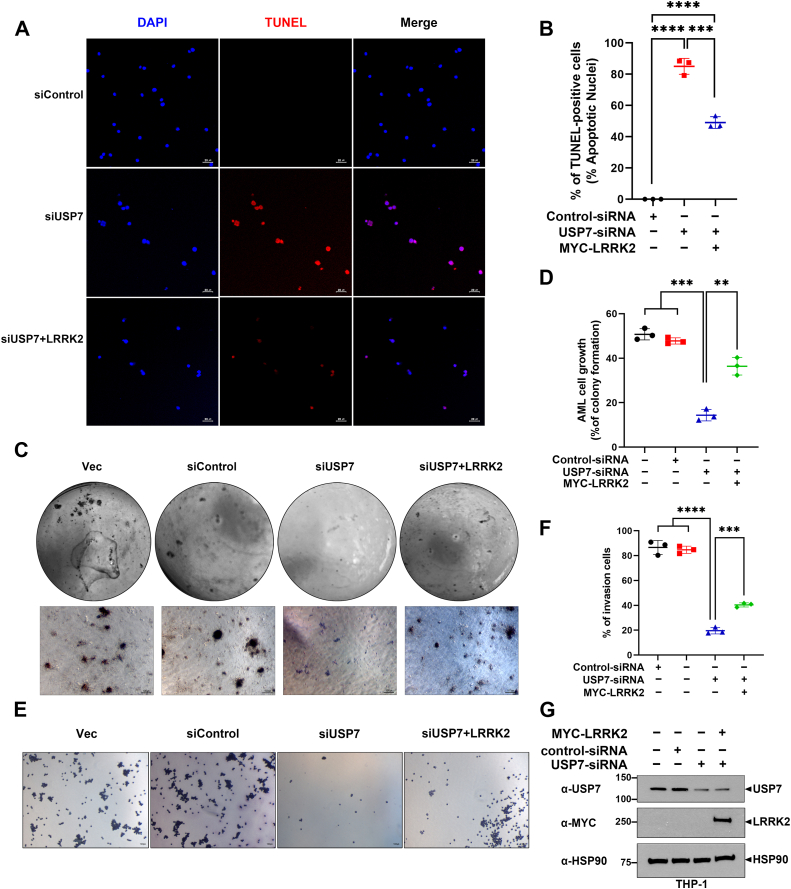
**Inhibition of AML cell growth by *USP7*-knockdown is partially restored by LRRK2 overexpression.***A*, *C*, and *E*, where indicated, THP-1 cells were transfected for 48 h with control siRNA or *USP7*-siRNA alone or together with Myc-LRRK2. *A*, after cell fixation with 2% paraformaldehyde and permeabilization, TUNEL reaction was performed with enzyme buffer containing TMR *red*. Fluorescence microscopy was then used to detect TMR *red* signals (*red*). DAPI (*blue*) was used for nuclear staining. *B*, the proportion of cells positive for TMR *red* signal was calculated. The results are presented as the mean ± SD from three independent experiments (∗∗∗∗*p* ≤ 0.0001; ∗*p* ≤ 0.05). *C*, the results of the colony formation assay are shown. After transfection with siRNA or DNA plasmid, cells were then cultured for 21 days in an agarose: media (1:1) mixture. Afterward, colonies were stained with 0.01% *crystal violet* and observed under a microscope. *D*, the colony formation ratio relative to the total area was quantified and expressed as a percentage. The results are presented as the mean ± SD from three independent experiments (∗∗∗*p* ≤ 0.001; ∗*p* ≤ 0.05). *E*, cell invasion assay results are shown. After 24 h of cell incubation in the inner chamber of a trans-well, cells that invaded into the lower chamber were stained with 0.05% crystal violet and observed under a microscope. *F*, the percentage of cells that invaded from the inner chamber of the trans-well to the bottom chamber was quantified. The results are presented as the mean ± SD from three independent experiments (∗∗∗∗*p* ≤ 0.0001; ∗∗∗*p* ≤ 0.001). *G*, THP-1 cells were transfected for 48 h with control siRNA or *USP7*-siRNA alone or together with Myc-LRRK2. Cell lysates were then subjected to immunoblotting with the indicated antibodies. HSP90 served as a loading control. AML, acute myeloid leukemia; DAPI, 4′, 6-diamidino-2-phenylindole; LRRK2, leucine-rich repeat kinase 2; USP7, ubiquitin-specific protease 7.

Next, we investigated the effect of siRNA-mediated *USP7*-knockdown on AML cell death. THP-1 cells were transfected with *USP7*-siRNA alone or together with Myc-LRRK2 for 48 h. Analysis of TUNEL-positive cells using fluorescence microscopy revealed that *USP7*-knockdown alone led to a significant increase in apoptosis (Fig. 7*A*), whereas co-expression of LRRK2 and *USP7*-siRNA partially reduced apoptosis (Fig. 7*B*).

We then examined the impact of *USP7*-knockdown and/or LRRK2 overexpression on AML cell growth by performing a colony formation assay. THP-1 cells were subjected to *USP7*-knockdown for 48 h, with or without Myc-LRRK2 transfection, and then cultured for 21 days. Colonies were stained with 0.01% crystal violet. Microscopic analysis of the THP-1 cells showed that *USP7*-knockdown led to a significant reduction in colony formation (Fig. 7*C*), while co-expression of LRRK2 partially rescued colony formation (Fig. 7*D*). We also investigated the effect of *USP7*-knockdown and/or LRRK2 overexpression on AML cell invasion using a trans-well assay. As shown in Figure 7, *E* and *F*, *USP7*-knockdown significantly decreased THP-1 cell invasion into the lower chamber, but this effect was partially restored by co-expression of Myc-LRRK2 (Fig. 7, *E* and *F*).

Finally, to confirm that the phenotypic changes were accompanied by the expected modulation of protein expression, we performed immunoblot analysis following *USP7*-knockdown and/or LRRK2 overexpression. THP-1 cells transfected with *USP7*-siRNA alone or together with Myc-LRRK2 showed efficient USP7 depletion and robust LRRK2 overexpression, validating the effectiveness of siRNA knockdown and transfection conditions (Fig. 7*G*).

Taken together, these results suggest that *USP7*-knockdown inhibits THP-1 cell proliferation, reduces AML cell growth, increases apoptosis, and suppresses the oncogenic potential of AML cells. However, all these effects can be partially rescued by LRRK2 overexpression.

#### USP7 potentiates LRRK2-mediated HDAC3 phosphorylation

Regarding LRRK2 substrates, we previously demonstrated that LRRK2 directly phosphorylates HDAC3, enhancing its activity and influencing histone modifications epigenetically (2). Interestingly, depending on the cancer type, HDAC3 is known to either promote oncogenesis (24, 25) or act as a tumor suppressor (26, 27). Based on our findings and others' research, we hypothesized that if LRRK2 degradation is inhibited and its stability is increased through the deubiquitinase action of USP7, the downstream target HDAC3 would be affected, further promoting its oncogenic role in AML.

**Figure 8:**
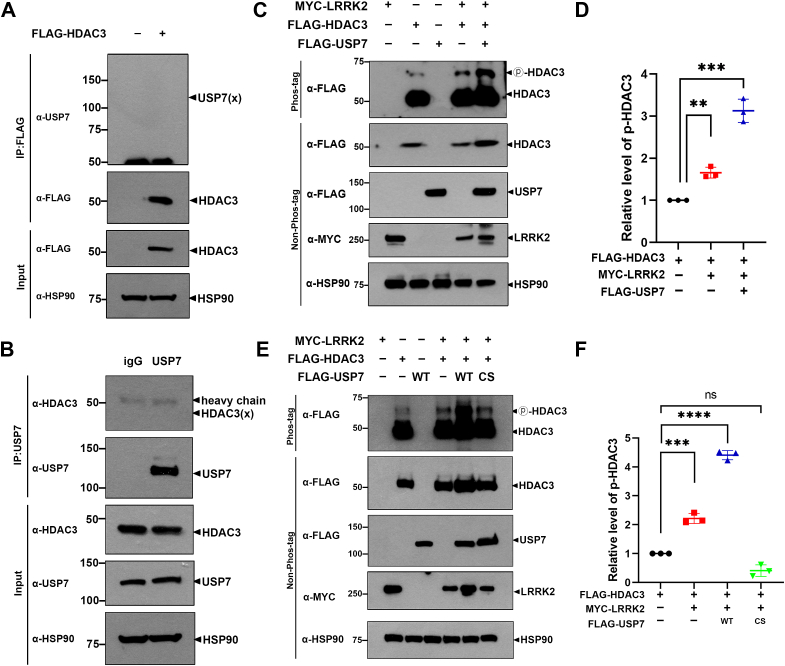
**USP7 potentiates the LRRK2-mediated HDAC3 phosphorylation.***A*, HEK293 cells were transfected with plasmid encoding Flag-HDAC3-WT for 24 h, followed by immunoprecipitation using anti-Flag antibody. Cell lysates were then immunoblotted with the indicated antibodies. *B*, immunoprecipitation of HEK293 cell lysates was performed using anti-USP7 antibody or IgG (control), followed by immunoblotting with the indicated antibodies. *C*, where indicated, HEK293 cells were transfected for 24 h with plasmids encoding Flag-HDAC3, Myc-LRRK2, or Flag-USP7-WT alone or in combination. Cell lysates were immunoblotted using anti-Flag antibody on a 40 mM Phos-tag gel. For the non-Phos-tag gel, immunoblotting was performed with the indicated antibodies. *D*, the relative phosphorylation levels of HDAC3 were quantified, and the results are presented as the mean ± SD from three independent experiments (∗∗∗*p* ≤ 0.001; ∗∗*p* ≤ 0.01). *E*, where indicated, HEK293 cells were transfected for 24 h with plasmids encoding Flag-HDAC3, Myc-LRRK2, Flag-USP7-WT, or Flag-USP7 CS alone or in combination. Cell lysates were immunoblotted using anti-Flag antibody on a 40 mM Phos-tag gel. For the non-Phos-tag gel, immunoblotting was performed with the indicated antibodies. *F*, the relative phosphorylation levels of HDAC3 were quantified, and the results are presented as the mean ± SD from three independent experiments (∗∗∗∗*p* ≤ 0.0001; ∗∗∗*p* ≤ 0.001; NS, not significant). HSP90 served as a loading control. HDAC, histone deacetylase; HEK293, human embryonic kidney 293; IgG, immunoglobulin G; LRRK2, leucine-rich repeat kinase 2; USP7, ubiquitin-specific protease 7.

To investigate this, we first tested whether USP7 interacts with HDAC3. After transfecting HEK293 cells with Flag-HDAC3, we examined the interaction with endogenous USP7. co-IP analysis showed that endogenous USP7 does not bind to Flag-HDAC3 (Fig. 8*A*). Another co-IP experiment, conducted without exogenous DNA transfection, confirmed no direct interaction between endogenous USP7 and HDAC3 (Fig. 8*B*).

Next, we compared phospho-HDAC3 levels in the presence or absence of LRRK2 and/or USP7 using Phos-tag SDS-PAGE. As expected and consistent with our previous findings, the intensity of the phospho-HDAC3 band increased by over 50% in the presence of LRRK2 compared to the mock-transfected control (Fig. 8, *C* and *D*). In addition, cells co-transfected with LRRK2 and USP7-WT showed a 2.8- to 3.1-fold increase in phospho-HDAC3 levels (Fig. 8, *C* and *D*). The catalytic-inactive USP7 mutant (USP-CS) did not affect HDAC3 phosphorylation (Fig. 8, *E* and *F*).

Collectively, these results suggest that USP7 positively regulates HDAC3 phosphorylation through upregulation of LRRK2. We propose that the oncogenic effect of USP7 in AML is partly due to its positive impact on HDAC3 activity and consequent epigenetic regulation.

#### USP7 enhances the levels of WT LRRK2 and two PD-related mutants (D1994A and R1441C), but not the LRRK2-G2019S mutant

Although there is limited evidence and some inconsistencies in research findings, recent studies have suggested that two familial PD-linked mutants, LRRK2-G2019S and LRRK2-R1441C, not only contribute to PD development but may also be associated with an increased incidence of certain cancers, such as colon and gastrointestinal cancers (28, 29). Based on these reports and our current findings on the oncogenic activity of USP7 and LRRK2 in AML cells, we investigated whether USP7 could modulate the expression levels of three PD-associated LRRK2 mutants (G2019S, R1441C, and D1994A). HEK293 cells were transfected for 24 h with plasmids encoding Myc-LRRK2-WT, Myc-LRRK2-G2019S, Myc-LRRK2-D1994A, or Myc-LRRK2-R1441C, either alone or in combination with Flag-USP7-WT. Immunoblotting of cell lysates showed that USP7-WT significantly increased the expression of LRRK2-WT, LRRK2-D1994A, and LRRK2-R1441C mutants. However, USP7 had no significant effect on the LRRK2-G2019S level (Fig. S6, *A* and *B*).

These results suggest that USP7 enhances the levels of WT LRRK2 through UPS regulation but has minimal impact on the PD-linked LRRK2-G2019S mutant.

## Discussion

LRRK2 plays a pivotal role in regulating various cellular processes, such as mitochondrial function and protein trafficking within the endoplasmic reticulum, and is widely expressed across a diverse array of tissues. For example, LRRK2 is expressed in the brain, kidney, lung, spleen, and immune cells including lymph nodes and white blood cells (30). Recent studies have also shown that dysregulation of the *LRRK2* gene is associated with the tumor development of various tissues, particularly lung, renal tissue, thyroid, and bile duct. For example, *LRRK2*-knockdown induces apoptosis and suppresses the progression of lung cancer through modulation of TLR4/NF-κB signaling pathway and the NLRP3 inflammasome (10). In clear cell renal cell carcinoma, LRRK2 expression is significantly elevated, and higher levels of LRRK2 expression correlate with increased clear cell renal cell carcinoma progression (6). In thyroid cancer, *LRRK2*-knockdown inhibits JNK signaling, leading to cell cycle arrest and reduced cell migration and proliferation (31). Similarly, downregulation of LRRK2 in bile duct cancer (intrahepatic cholangiocarcinoma) suppresses intrahepatic cholangiocarcinoma proliferation and metastasis (9). In addition, the present study suggests that LRRK2 plays an oncogenic role in AML.

AML is a heterogeneous hematologic malignancy characterized by the rapid proliferation of abnormal myeloid precursor cells in the bone marrow and peripheral blood, which impairs normal hematopoiesis. It is typically associated with mutations in genes that regulate cell differentiation, proliferation, and apoptosis, leading to the accumulation of immature white blood cells (32). Among the genes currently being explored for targeted therapies are *FLT3*, *NPM1*, and *IDH1/2*, which are linked to high-risk factors. Despite ongoing research, AML remains an incurable disease, with relapses and other complications still prevalent (33, 34, 35). Moreover, several USP enzymes play a crucial role in the pathogenesis of AML. For instance, USP10 prevents the degradation of FLT3 kinase in AML cells, thereby stabilizing it and increasing AML progression (36). In addition, inhibition or deletion of USP15 has been found to impair leukemic progenitor function and reduce the viability of AML cells (37). In this context, the current study proposes that USP7 may play an oncogenic role in AML, and the positive regulatory relationship between USP7 and LRRK2 could potentially serve as a novel target for prognostic or therapeutic strategies in AML.

Recent studies have highlighted the involvement of USP7 in regulating the expression of various cancers. Depending on the type of cancer, USP7 can act either as an oncogene or a tumor suppressor. In CLL, inhibition of USP7 induces apoptosis and suppresses CLL progression (38). USP7 also promotes breast carcinoma by stabilizing the histone demethylase PHF8 (39), while in non—small cell lung cancer, downregulation of USP7 reduces cell metastasis and invasion (40). Furthermore, in epithelial ovarian cancer, USP7 has been found to positively regulate the vitality and invasion of ovarian cells (41). As demonstrated in recent studies, including the present one, USP7 would be involved in the development of various cancers, including AML. These findings, like those of other USPs or DUBs, highlight the crucial role of deubiquitination of multiple targets mediated by USP7 in various cellular growth and regulatory processes. Disruption of this balance can contribute to the development of various cancers, thus underscoring the functional significance of USP7.

Considering diverse physiological roles of LRRK2 and its close link to the progression of PD as well as cancer development, extensive research has been instrumental to explore the functional regulation and stability of LRRK2. Previous studies have shown that several enzymes control the activity of LRRK2 through post-translational modifications, including the phosphorylation by multiple kinases, such as casein kinase 1α (CK1α), IKK, and PKA (42, 43). For example, phosphorylation of LRRK2 by casein kinase 1α regulates trans-Golgi clustering *via* differential interaction with ARHGEF7 (44). In addition, PP1 and PP2A phosphatases dephosphorylate LRRK2, reversely modulating the activity or biochemical property of LRRK2 (45). LRRK2 also becomes to be a target of ubiquitination. For example, two ubiquitin E3 ligases, such as TRIM1 and CHIP, induce the degradation of LRRK2 through UPS (4, 5). With regard to the proteolysis, LRRK2 is also degraded in lysosomes by chaperone-mediated autophagy (3). Like phosphorylation, many targets of protein ubiquitination could be dynamically regulated by deubiquitination. Nevertheless, no DUBs have been identified that enhance the stability of LRRK2 through deubiquitination. Therefore, it is crucial to identify DUB(s) that positively modulate the stability of LRRK2. Here, we have identified USP7 for the first time, specifically targeting to LRRK2 and causing its accumulation through deubiquitination.

Similar to LRRK2, USP7 performs a wide range of cellular functions. USP7 has been shown to catalyze the deubiquitination of MyD88, claspin, p53, ICN1, SRSF6, TRIP13, and histone H2A. (12). For instance, USP7 modulates immune responses by upregulating MyD88 and increasing proinflammatory factors (12, 46). It also markedly prolongs the half-life of claspin, an adaptor protein that facilitates the ataxia-telangiectasia, which in turn increases the magnitude and duration of DNA damage response upon genotoxic stress. Moreover, USP7 also assists USP47 in deubiquitinating the NLRP3 inflammasome, thereby enhancing its activation (14). The present study identifies LRRK2 as a novel substrate of USP7, promoting the growth of AML cells.

In addition to genetic factors, epigenetic alterations have fundamental functions in cancer progression characterized by reversibility and susceptibility to external factors. Among multiple histone deacetylases (HDACs), HDAC3 is an epigenetic drug target which is currently marked as a potential therapeutic strategy to combat various cancers. For example, a critical role for HDAC3 was identified in *KRAS*-mutant lung cancer (47). In colorectal cancer, overexpression of HDAC3 causes a decrease in the expression of p21. Inhibition of HDAC3 induces the acetylation process of p53 and p21 expression that results in G1 phase arrest in triple-negative breast cancer. The expression of HDAC3 is also significantly correlated with the B7-H1 expression in gastric cancer (48). Moreover, HDAC3 can repress the metastatic potential of colorectal cancers by binding to the promoter sequences of Runx-2 (49).

Regarding the putative role of LRRK2 to promote tumor progression, we previously revealed that LRRK2 phosphorylates HDAC3 at S424, thereby stimulating HDAC activity. As a result, LRRK2 exacerbated neuronal cytotoxicity through phosphorylation of HDAC3 and histone deacetylation. Considering the close link between HDAC3 and its oncogenic action in many types of cancer, we could speculate that the USP7-mediated blockade of LRRK2 degradation may also contribute the tumor development, including AML. This speculation was further supported by other study that PD-L1 is regulated by HDAC3, with its expression varying depending on cancer type or cell context. Based on these reports, we next aimed to investigate how USP7-mediated elevation of LRRK2 level affects the phosphorylation of HDAC3. If that is the case, we could further hypothesize that USP7- or LRRK2-mediated regulation of HDAC3 could be directly linked to AML cell growth. As expected, our results showed that UPS7 increases HDAC3 phosphorylation through the quantitative regulation of LRRK2, suggesting that the upregulation of LRRK2 by USP7 may contribute to the AML, which would be interesting to verify by further experiment.

Although an inverse relationship between PD and cancer has been well documented, the association between cancers and genetic forms of the LRRK2 gene remains insufficiently understood. The analysis of cancer prevalence in PD patients with R1441G or G2019S mutations in LRRK2 (50) revealed no significant increase in cancer incidence associated with these mutations. In addition, a slightly higher, but not statistically significant, prevalence of non-skin cancers was found in the G2019S group. In contrast, recent clinical study has shown that G2019S carriers are at an elevated risk for various cancers, including colon cancer. The present finding yields different results: unlike wild-type LRRK2 and its two PD-associated mutants (*i.e.*, LRRK2-D1994A and LRRK2-R1441C), the intracellular level of LRRK2-G2019S mutant is unaffected by USP7. These results suggest that LRRK2-G2019S mutant may influence the growth of AML cells in a distinct manner. Furthermore, more systematically designed studies are needed to evaluate this association in detail and determine whether there is an increased cancer risk in this population.

In conclusion, the overall study demonstrated that USP7 promotes the deubiquitination of LRRK2 as an upstream regulator, thereby preventing the degradation of LRRK2 through UPS and positively regulating its level. Furthermore, we demonstrated that USP7 regulates LRRK2 level in AML cells, influencing the growth and oncogenic potential of AML cells. Although our research was conducted exclusively *in vitro* using AML cell lines, future *in vivo* studies using immunodeficient mouse models or samples from AML patients will be essential to further validate and extend our findings. If confirmed, this finding could verify USP7 and its downstream target of LRRK2 as key contributor to AML pathogenesis, positioning USP7 as a potential causative factor and a promising target for novel anticancer therapies.

## Experimental procedures

### Materials

Dulbecco's modified Eagle medium (DMEM), Fetal Bovine Serum (FBS), Penicillin-Streptomycin (P/S), Roswell Park Memorial Institute 1640 Medium (RPMI 1640), and McCoy’s modified 5A medium were purchased from Thermo Fisher Scientific. Polyethyleneimine reagent and Lipofectamine 2000 were purchased from Invitrogen. TransIT-X2 Dynamic Delivery System for Plasmid DNA reagents were purchased from Mirus Bio. Alexa Fluor 488-conjugated mouse IgG, Alexa Fluor 594-conjugated rabbit IgG, and ProLong Gold Antifade Mountant with 4′, 6-diamidino-2-phenylindole (DAPI) were purchased from Invitrogen. HBX19818 (HY-17540) was purchased from MedChemExpress (MCE), and P5091 (ab144602) was purchased from Abcam. Protein A-Sepharose beads and Ni-NTA and Co-NTA agarose beads were purchased from GE Healthcare Life Sciences. Enhanced chemiluminescence (ECL) reagents were purchased from AbClon. Western Bright ECL was purchased from Advansta. Normal mouse IgG and normal rabbit IgG were purchased from Santa Cruz Biotechnology. Skim milk was purchased from Merck. Duolink PLA assay kit (DUO92101) was purchased from Sigma-Aldrich. SuperSep Phos-tag 7.5% gel was purchased from FujiFilm Wako. TRIzol reagent (15596-026) was purchased from Invitrogen. SYBR Green PCR Master Mix (4367659) was from Thermo Fisher Scientific, and TOPscript cDNA synthesis kit (EZ005S) was purchased from Enzynomics. The e-MYCO *Mycoplasma* Detection Kit (ver 2.0; 25235) was purchased from iNtRON Biotechnology. Mouse monoclonal anti-Flag (F3165) and mouse polyclonal anti-Myc (sc-40) antibodies were purchased from Sigma-Aldrich. Mouse anti-HSP90 (sc-13119), mouse anti-GFP (sc-9996), anti-β-actin (sc-47778), and anti-GAPDH (sc-32233) antibodies were purchased from Santa Cruz Biotechnology. Anti-α-tubulin (GTX628802) antibody was from GeneTex. Horseradish peroxidase-conjugated anti-rabbit and anti-mouse secondary antibodies were purchased from EMD Millipore. Rabbit monoclonal anti-HDAC3 (ab137704) and recombinant rabbit anti-LRRK2 (ab133474) antibodies were purchased from Abcam. Polyclonal rabbit anti-USP7 (GTX125894) and monoclonal mouse anti-USP7 (GTX631108) antibodies were purchased from GeneTex.

### DNA constructs and RNA interference

The plasmids encoding Flag-tagged human WT USP7 (pCMV2-Flag-USP7-WT) and its catalytic-inactive USP7-C223S mutant, in which the Cys-223 residue is substituted with serine (pCMV2-Flag-USP7-CS), as well as the plasmids encoding Flag-tagged four deletion mutants of USP7 (Flag-USP7^1-208^, Flag-USP7^206-560^, Flag-USP7^560-776^, and Flag-USP7^776-1102^; denoted as D1–D4), were kindly provided by J.W. Kim (Inha University). The plasmids encoding Myc-tagged WT LRRK2 (pcDNA3-6xHis/Myc-LRRK2-WT), LRRK2-G2019S (pcDNA3-6xHis/Myc-LRRK2-GS), and LRRK2-D1994A mutant (pcDNA3-6xHis/Myc-LRRK2-DA) were kindly provided by B.D. Lee (Kyung Hee University, Seoul, Korea). The plasmid encoding Flag-tagged WT HDAC3 (pcDNA3-3xFlag-HDAC3-WT) was provided by E. Seto (The George Washington University, Washington D.C., USA). To generate DNA construct encoding the Myc-LRRK2-R1441C mutant, site-directed mutagenesis was performed using the QuickChange XL site-directed mutagenesis kit (Stratagene Inc) with pcDNA3-6xHis/Myc-LRRK2 as a template. All DNA construct sequences were confirmed by DNA sequencing (Bionics Inc). The siRNAs for USP7 and control scrambled siRNA (#51-01-14-04) were purchased from IDT Korea. The USP7-specific siRNA #1 duplex sequences were as follows: 5′-AUCAGCAGCUUAAGAUGAAAAUCAC(dTdT)-3′ (sense) and 5′-GUGAUUUUCAUCUUAAGCUGCUGAUAG(dTdT)-3′ (antisense). The USP7-specific siRNA #2 duplex sequences were 5′-GUUUGGCUUCUCUGUAUCUAUUGAC(dTdT)-3′ (sense) and 5′-GUCAAUAGAUACAGAGAAGCCAAACUG(dTdT)-3′ (antisense).

### Cell culture and DNA transfection

HEK293 cells were maintained in DMEM supplemented with 10% FBS and 100 U/ml P/S. Human neuroblastoma SH-SY5Y cells were maintained in a 1:1 mixture of DMEM and F12 media, supplemented with 10% FBS and 100 U/ml P/S. AML cells, including HL-60 and HEL cells, were kindly provided by S.S. Yoon (Seoul National University School of Medicine, Seoul, Korea); THP-1 cells by S.H. Park (Seoul National University, Seoul, Korea); MV4-11 and MOLM13 cells by J.W. Jung (Yonsei University College of Medicine, Seoul, Korea); and U937 cells by B.Y. Park (Yonsei University, Seoul, Korea). SK-BR-3 cells were kind gift from S.H. Kang (Yonsei University College of Medicine, Seoul, Korea). All AML cells were maintained in RPMI 1640 medium supplemented with 10% FBS and 100 U/ml P/S. Prostate cancer PC-3 cells were also maintained in DMEM medium with 10% FBS and 100 U/ml P/S. Human breast cancer SK-BR-3 cell line was maintained in McCoy’s 5A medium supplemented with 10% FBS and 100 U/ml P/S. All cells were grown at 37 °C in 5% CO_2_. Adherent cells were transfected using polyethyleneimine reagent, Lipofectamine 2000, or Mirus reagent, while suspension cells were transfected using the 4D-Nucleofector X Unit, according to the manufacturer’s instructions.

### Co-immunoprecipitation and immunoblot analysis

Cell lysates were collected by scraping, and the debris was extensively washed with ice-cold phosphate-buffered saline (PBS). The cells were then mixed with 1% Nonidet P-40 (NP40) lysis buffer (50 mM Tris, pH 7.5, 1% Nonidet P-40, 150 mM NaCl, and 10% glycerol) containing a protease inhibitor cocktail (1 mM NaF, 1 μg/ml leupeptin, 1 μg/ml aprotinin, 1 mM Na_3_VO_4_, and 0.2 mM phenylmethylsulfonyl fluoride), incubated on ice for 10 min, sonicated at 45 Hz for 7 s, and then centrifuged at 13,000 rpm for 15 min at 4 °C. For immunoprecipitation, 500 to 1000 μg of cell lysates were incubated with approximately 1 μg of the appropriate antibodies overnight at 4 °C with gentle rotation. The next day, the samples were incubated with protein A-Sepharose beads for 2 h at 4 °C, washed extensively with 500 μl NP40 lysis buffer supplemented with the protease inhibitor cocktail, and then eluted in 2× sample buffer by boiling for 5 min. The samples were separated by SDS-PAGE, transferred to a nitrocellulose membrane (Whatman, GE Healthcare Life Sciences), and blocked for 1 h at room temperature in 1× TBST buffer (20 mM Tris, pH 7.5, 137 mM NaCl, and 0.1% Tween 20) with 5% nonfat skim milk. The membranes were then incubated with the appropriate primary antibody overnight at 4 °C. After washing with 1× TBST, the membranes were incubated with horseradish peroxidase-conjugated secondary IgG for 2 h at room temperature. The protein bands were detected using ECL reagents (AbClon) or Western Bright reagent (Advansta), following the manufacturer’s instructions.

### Quantitative real-time PCR

Total RNA was extracted from various AML cell lines using TRIzol reagent (Thermo Fisher Scientific), followed by on-column DNase digestion using the RNeasy Mini Kit (Qiagen). Complementary DNA (cDNA) was synthesized from 1 μg of total RNA using the TOPscript cDNA Synthesis Kit (Enzynomics) with gene-specific primers for LRRK2 (forward: 5′-AGCAAGGGACAGGCTGAAGTTG-3′; reverse: 5′-GCAGGCTTTGCGTTGCTTCTCA-3′) and USP7 (forward: 5′-GTCACGATGACGACCTGTCTGT-3′; reverse: 5′-GTAATCGCTCCACCAACTGCTG-3′). qRT-PCR was performed using SYBR Green PCR Master Mix (Thermo Fisher Scientific) on a CFX Duet Real-Time PCR Detection System (Bio-Rad), and relative expression levels were normalized to GAPDH mRNA for each sample.

### Confocal microscopic analysis

Cells were seeded onto poly-L-lysine-coated cover slips in 6-well plates. After DNA transfection, the cells were washed three times with PBS (pH 7.4) and fixed with 3.7% formaldehyde for 10 min. Following fixation, cells were permeabilized with 0.1% Triton X-100 for 10 min, blocked with 1% bovine serum albumin in TBST for 1 h at room temperature, and immunostained using rabbit polyclonal anti-LRRK2 and/or rabbit polyclonal anti-USP7 antibodies. The samples were then incubated with Alexa Fluor 488- or Alexa Fluor 594-conjugated anti-IgG antibodies. Microscopic images were obtained using the LSM 980 confocal microscope (Carl Zeiss) and processed with the Zeiss LSM Image Browser (Carl Zeiss).

### Phospho-tag SDS-PAGE assay

After 24 h of DNA transfection, cells were washed three times with ice-cold PBS and lysed on ice using lysis buffer containing 0.1% NP-40, 50 mM Tris (pH 7.4), 150 mM NaCl, 10% glycerol, 0.2 mM phenylmethylsulfonyl fluoride, and a protease inhibitor cocktail. Cell lysates were separated on a 10% SDS-PAGE gel containing 40 mM Phos-tag (Wako Pure Chemical Industries). Phos-tag immunoblotting for phosphorylated HDAC3 was performed according to the manufacturer’s instructions. The phosphorylation level of USP7 was analyzed using a SuperSep Phos-tag 7.5% gel under conditions with or without LRRK2 overexpression, according to the manufacturer’s instructions.

### *In vitro* Ni^2+^/Co^2+^ pull-down assay

HEK293 cells were transfected for 24 h with a plasmid encoding six consecutive His-tagged LRRK2. After three washes with 1× PBS (pH 7.4), the cells were lysed using ice-cold NP40 buffer containing a protease inhibitor cocktail. The cell lysates were subjected to sonication at 45 Hz, followed by centrifugation at 13,000 rpm for 15 min, and the supernatant was collected. The samples were incubated with Ni-NTA resin (Thermo Fisher Scientific) at 4 °C with gentle rotation for 1 h. After centrifugation, the pellet was collected and washed with the binding buffer (50 mM Na_3_PO_4_, pH 8.0, 500 mM NaCl, 0.5% Triton X-100, and 10% glycerol) containing 10 mM imidazole at 4 °C for 20 min with gentle rotation. The resin was washed three times with 1× wash buffer containing 25 mM imidazole and eluted with an elution buffer containing 250 mM imidazole. The eluted proteins were analyzed by SDS-PAGE after boiling with 2× sample buffer. In addition, the pull-down protocol using Co^2+^-containing TALON magnetic beads (Takara) was performed according to the manufacturer’s instructions.

### TUNEL assay

After transfection for 24 or 48 h with *USP7*-siRNA or plasmid encoding Myc-LRRK2, or treatment with the chemical inhibitor of UPS7 alone or in combination, THP-1 cells were centrifuged at 13,000 rpm. The pellet was resuspended in 1 × NP40 buffer containing a protease inhibitor cocktail and incubated on ice for 15 min. Following incubation, the cells were lysed by sonication at 45 Hz, and the cell lysates were collected. The cell suspension was fixed with 2% paraformaldehyde in PBS (pH 7.4) for 1 h, followed by permeabilization with 0.1% Triton X-100 in PBS and 0.1% sodium citrate for 5 min. The cells were then incubated for 1 h in TUNEL reaction buffer containing TMR red label solution and terminal deoxynucleotidyl transferase (TdT) enzyme. After centrifugation at 13,000 rpm, the samples were washed three times with 1 × PBS buffer, followed by counterstaining of the nuclei with DAPI. The samples were mounted on confocal 35 mm plates (Ibidi, GmbH) and analyzed using an LSM980 confocal microscope (Carl Zeiss). Images were acquired, and image processing was performed with Zeiss LSM Blue 3.0 Image Browser (Carl Zeiss).

### THP-1 cell growth assay

THP-1 cells were plated at a density of 1.5 × 10^5^ cells per 35 mm culture plate, subjected to DNA or siRNA transfection for 24 h, and treated with 20 μM P5091 or 10 μM HBX19818 for additional 24 h. The cells were transferred to a 96-well plate, and cell viability was assessed using the CCK8 assay kit according to the manufacturer's instructions. After adding the CCK8 reagent to each well, the cells were incubated for 2 h, and the absorbance change was measured at 460 nm using a plate reader. The viability of live cells was compared to the control to determine the THP-1 cell viability.

### LDH assay

THP-1 cell death was evaluated using an LDH Cytotoxicity Detection Kit (Takara). THP-1 cells were transfected with DNA for 24 h, followed by treatment with 20 μM P5091 or 10 μM HBX19818 for an additional 24 h. Cell-free culture media were then collected and used in the LDH assay, according to the manufacturer’s instructions. Two types of controls were used to measure LDH release. The maximum LDH release (referred to as “high control”) was determined by solubilizing the cells in 1% Triton X-100. Spontaneous LDH release (referred to as “low control”) was determined by incubating the cells in the medium alone. Absorbance was measured at 490 nm using a microplate reader. Cytotoxicity, proportional to the amount of dead AML cells, was calculated as a percentage of the control using the following formula: cytotoxicity = [(experimental value–low control)/(high control - low control)] × 100%.

### Soft agar colony formation assay

A soft agar assay was performed to evaluate the colony-forming ability of THP-1 suspension cells. For assay, a base layer was prepared on 35 mm plates by mixing 1% soft agar (micro agar, Bio-Rad) with RPMI 1640 culture medium at a 1:1 ratio and solidifying 2 ml of the solution. Transfected cells were resuspended in a 1:1 mixture of RPMI 1640 medium and 0.4% soft agar, and 2 ml of this suspension was then plated on top of the base layer. The cell density was adjusted to 1.5 × 10^5^ cells per plate. The agar was solidified by incubation at 4 °C for 15 min before being transferred to 37 °C for further incubation. Plates were maintained in a humidified atmosphere at 37 °C for 15 to 21 days. After incubation, cells were fixed for 1 h with 4% paraformaldehyde, washed three times with 1× PBS (pH 7.4), and stained with 0.01% crystal violet (Merck) for 30 min at room temperature. Plates were then washed again with PBS. The colonies were analyzed under a microscope, and images were captured. The proportion of colonies formed relative to the total area was quantified from the captured images.

### Invasion migration assay

The invasion and migration activity of THP-1 cells was detected using a 24-well trans-well chamber permeable supports with 8.0-μm pores (Corning). After 24 h of DNA or siRNA transfection in THP-1 cells, cells were resuspended in serum-free medium, and 1 × 10^5^ cells were added into the coated upper chamber. Complete RPMI 1640 medium containing 10% FBS and 100 U/ml P/S was added to the lower chamber, and the cells were incubated for 24 h at 37 °C in a 5% CO_2_ environment. The cells were then fixed with 4% formaldehyde for 30 min at room temperature, washed three times with 1× PBS, and stained with 0.05% crystal violet for additional 15 min at room temperature. The number of invading cells on the lower chamber was counted and analyzed under a light microscope.

### Proximity ligation assay

The PLA was conducted using the Duolink PLA assay kit (DUO92101, Sigma-Aldrich), following the manufacturer’s protocols. The PLA signals are shown in red, and the nuclei are displayed in blue (DAPI).

### Cell line authentication and *mycoplasma* test

All human cell lines used in this study were either obtained directly from certified repositories such as American Type Culture Collection and KCLB, or were kindly provided by laboratories as noted in the acknowledgments. Although we did not perform in-house reauthentication *via* STR or SNP profiling, all cell lines originated from sources certified to perform such authentication procedures. Cells were routinely tested every 2 months and confirmed to be mycoplasma-free using a PCR-based *mycoplasma* detection assay (e-MYCO *Mycoplasma* Detection Kit ver. 2.0, iNtRON Biotechnology, #25235), performed according to the manufacturer’s instructions.

## Statistical analysis

All statistical analyses were performed using an unpaired student’s t-tests and one-way ANOVA. All values are reported as mean ± SD of at last three independent experiments. The GelQuant.NET software (version 1.8.2; http://biochemlabsolutions.com) was used for measuring the Western blot gel intensities. All graphs were generated using Prism 9 software (version 9.5.0; GraphPad.Inc). Because of the limited sample sizes in our experiments, formal normality testing was not performed, and statistical outlier analysis was not applied. No data points were excluded unless otherwise stated.

## Data availability

All datasets are included within the manuscript or are available upon reasonable request from the corresponding author: Kwang Chul Chung (kchung@yonsei.ac.kr).

## Supporting information

This article contains supporting information.

## Conflict of interest

The authors declare that they have no conflicts of interest with the contents of this article.
