# Supplementary material for: Ubiquitin-specific protease 7 promotes the growth and oncogenic potential of acute myeloid leukemia cells through the deubiquitination and upregulation of LRRK2
Source: J Biol Chem. 2025 Sep 2;301(10):110675. doi: 10.1016/j.jbc.2025.110675 (PMC12510031; doi:10.1016/j.jbc.2025.110675)
Supplement: Supporting Figures [file mmc1.pdf]

Supporting information

Ubiquitin-specific protease 7 promotes the acute myeloid leukemia cell growth and its oncogenic potential through the deubiquitination and upregulation of LRRK2

Joon Hyung Park, and Kwang Chul Chung

Contents

1. **Figure S1**. LRRK2 binds to the TRAF and UBL-3, -4, and -5 domains of USP7.
2. **Figure S2**. The phospho-USP7 level is unaffected by LRRK2 overexpression.
3. **Figure S3**. Treatment of proteasome inhibitor, MG132, causes the accumulation of LRRK2 without affecting USP7 level.
4. **Figure S4**. Ni-pulldown analysis following co-IP demonstrates that USP7, but not its catalytically inactive mutant, reduces LRRK2 ubiquitination.
5. **Figure S5**. Treatment of AML cells with HBX19818, another selective inhibitor of UPS7, suppresses AML cell growth, and it is partially rescued by LRRK2 overexpression.
6. **Figure S6**. USP7 enhances the levels of wild-type (WT) LRRK2 and two PD-related (D1994 and R1441C) mutants, but not the LRRK2-G2019S mutant.

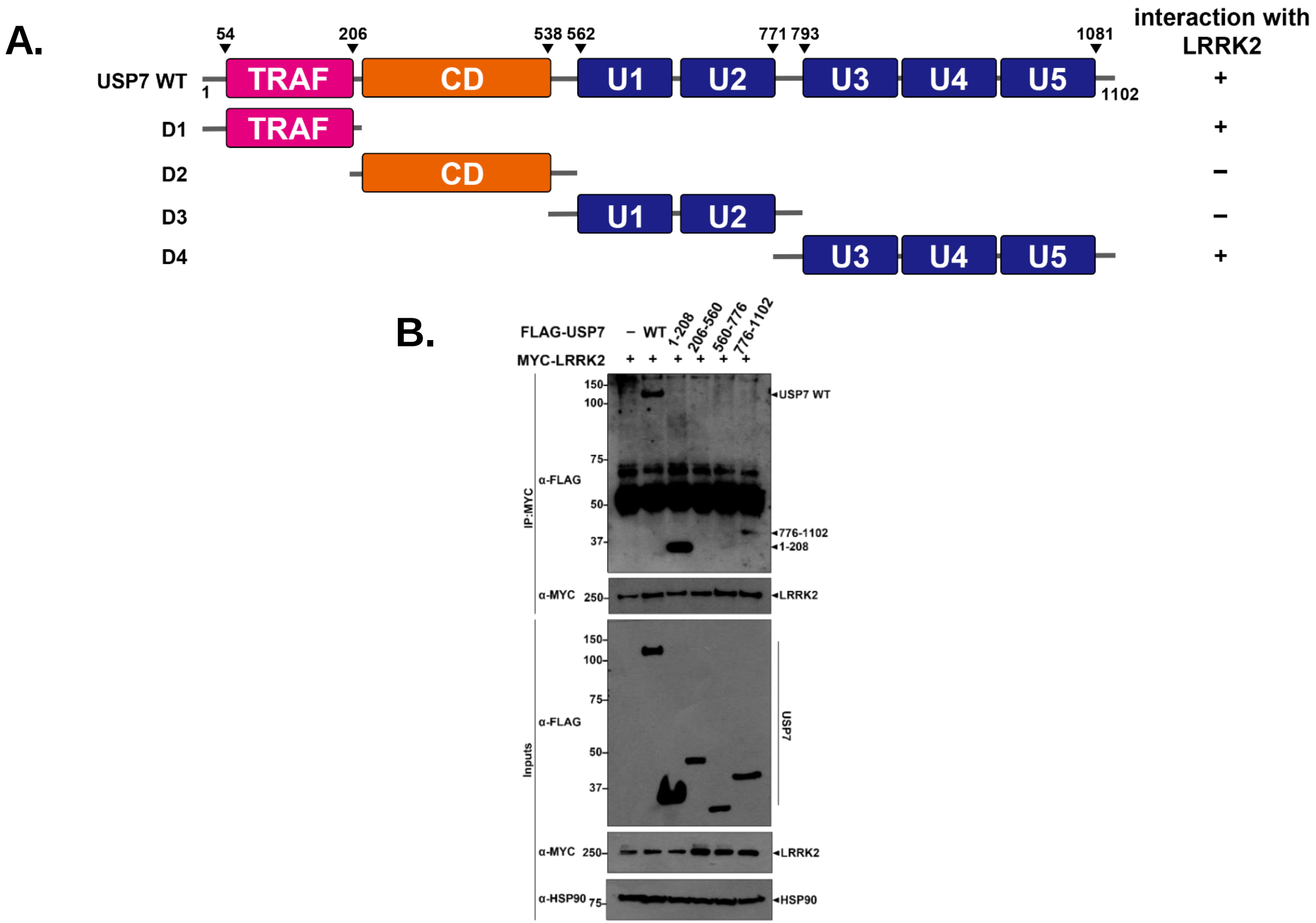

Figure S1. LRRK2 binds to the TRAF and UBL-3, -4, and -5 domains of USP7 (A) The schematic diagram of wild type USP7 and its 4 deletion mutants (D1-D4), showing TRAF domain (TRAF), catalytic domain (CD), and five UB-like subdomain (U1-U5). The summarized results of the co-IP for the interaction between LRRK2 and USP7 are shown on the right. ‘+' denotes binding, while ‘-' indicates no binding. (B) HEK293 cells were transfected for 24 h with Myc-LRRK2 or/and the indicated USP7-deletion mutant. Subsequently, cell lysates were immunoprecipitated using anti-Myc antibody, followed by immunoblotting with the indicated antibodies. HSP90 served as a loading control.

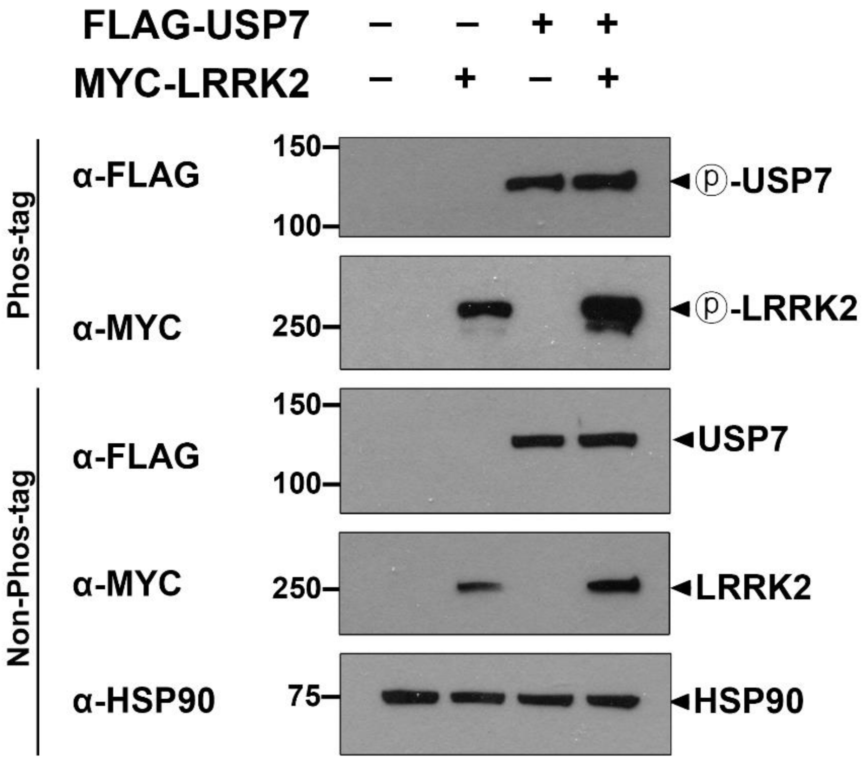

Figure S2. The phospho-USP7 level is unaffected by LRRK2 overexpression. As indicated, THP-1 cells were transfected for 24 h with plasmids encoding Myc-LRRK2 and/or Flag-USP7, either alone or in combination. Following DNA transfection, cell lysates were subjected to immunoblotting using anti-Flag or anti-Myc antibodies on a 50 mM Phos-tag SDS-PAGE gel to assess phosphorylation status of USP7 or LRRK2, respectively. For comparison, conventional SDS-PAGE (non-Phos-tag) was also used, and immunoblotting was performed with the indicated antibodies. HSP90 served as a loading control.

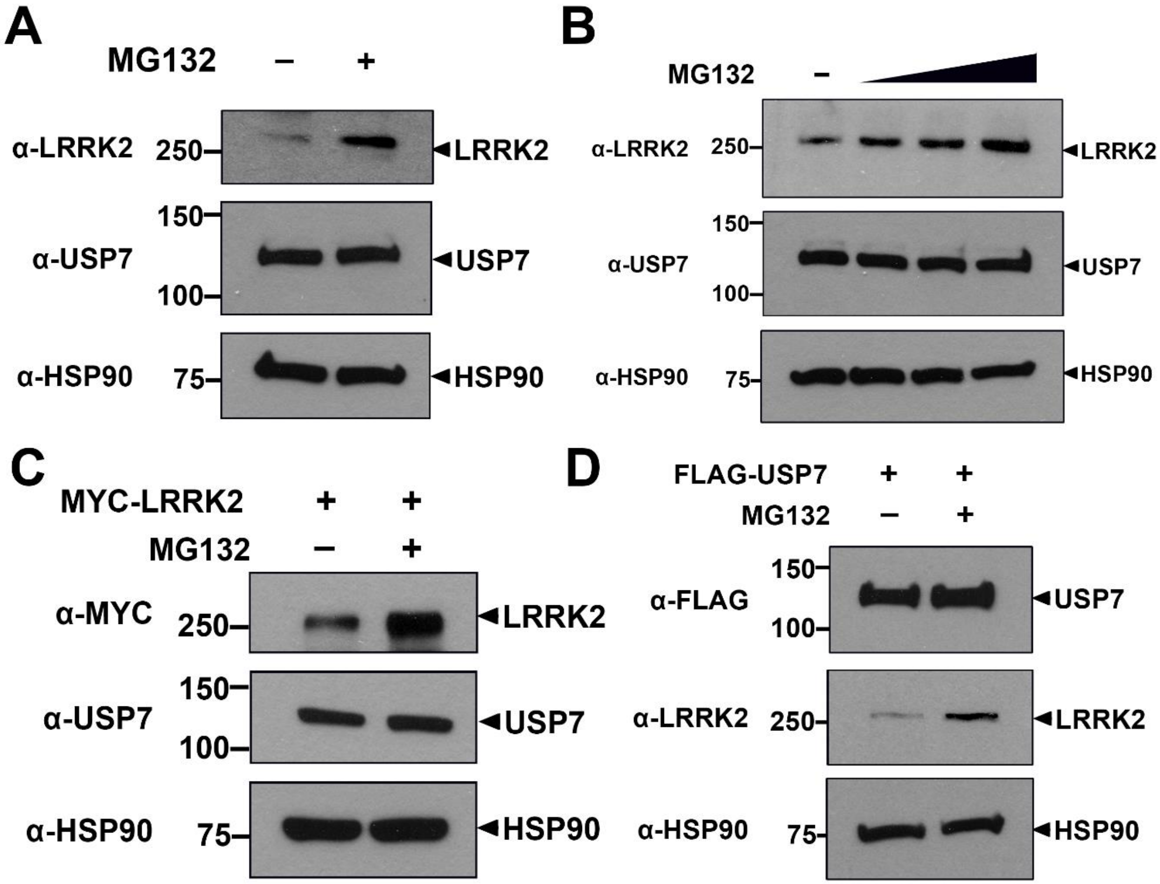

Figure S3. Treatment of proteasome inhibitor, MG132, causes the accumulation of LRRK without affecting USP7 level. (A) Where indicated, HEK293 cells were treated with 20 μM MG132 for 4 h, and cell lysates were subjected to immunoblotting with the indicated antibodies to assess endogenous LRRK2 and USP7 protein levels. (B) HEK293 cells were treated with increasing concentrations of MG132 (0, 5, 10, and 20 μM) for 4 h, followed by immunoblotting of cell lysates with the indicated antibodies to evaluate the dose-dependent effects on endogenous LRRK2 and USP7 levels. (C) HEK293 cells were transfected with a plasmid encoding Myc-LRRK2 for 24 h, followed by treatment with 20 μM MG132 for 4 h; cell lysates were then analyzed by immunoblotting using the indicated antibodies. (D) HEK293 cells were transfected with a plasmid encoding Flag-USP7 for 24 h, followed by treatment with 20 μM MG132 for 4 h; immunoblotting of cell lysates was performed with the indicated antibodies to assess LRRK2 and USP7 protein levels. HSP90 served as a loading control.

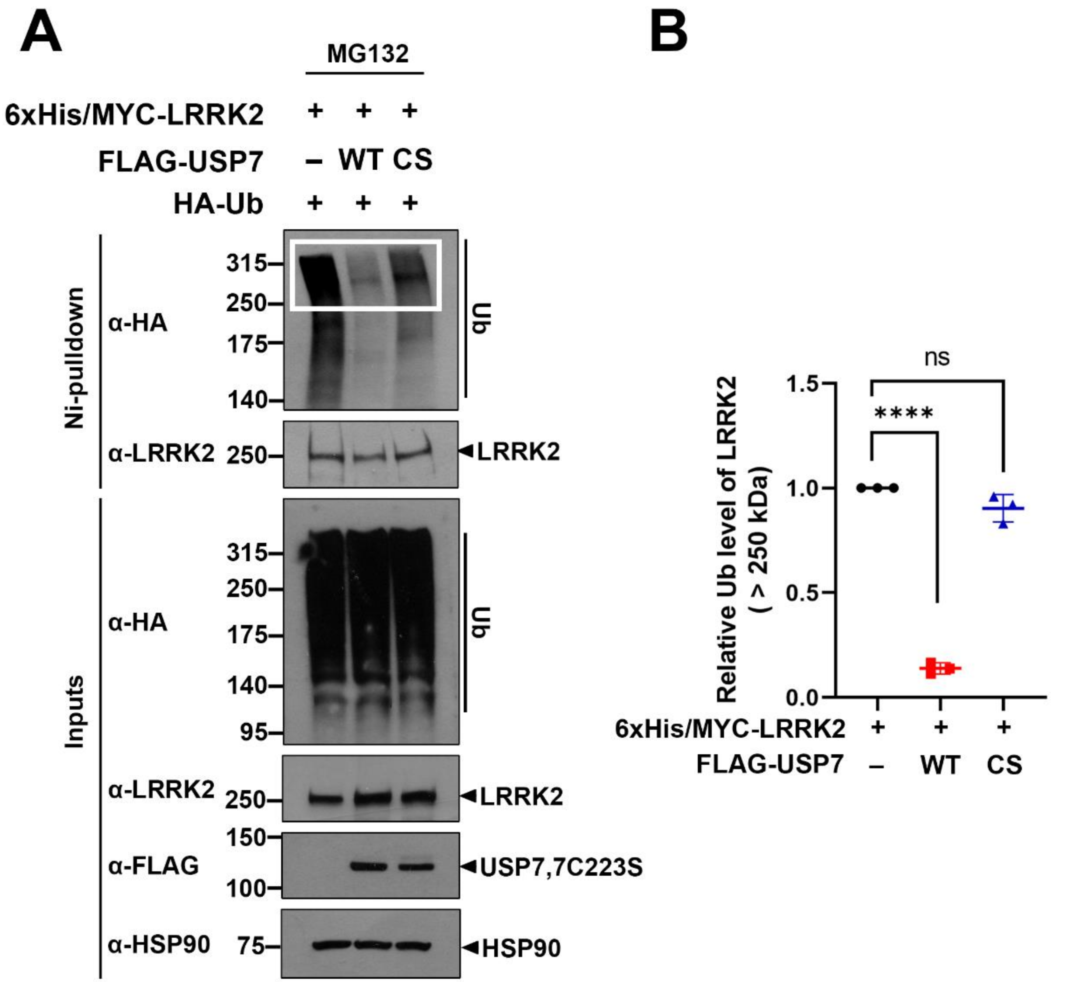

Figure S4. Ni-pulldown analysis following co-IP demonstrates that USP7, but not its catalytically inactive mutant, reduces LRRK2 ubiquitination. (A) As indicated, HEK293 cells were transfected for 24 h with plasmids encoding 6xHis/Myc-LRRK2 and HA-Ub, either alone or in combination with Flag-USP7-WT or Flag-USP7-CS. After transfection, cells were treated with 20 μM MG132 for an additional 4 h. Cell lysates were incubated with Ni^{2+}-NTA beads for 2 h, and bound proteins were eluted using imidazole. The eluates were then subjected to immunoprecipitation with an anti-LRRK2 antibody, followed by immunoblotting with the indicated antibodies. HSP90 was used as a loading control. **(B)** The relative levels of LRRK2 ubiquitination were quantified and are presented as the mean ± SD from three independent experiments (*****p* ≤ 0.0001; NS, not significant).

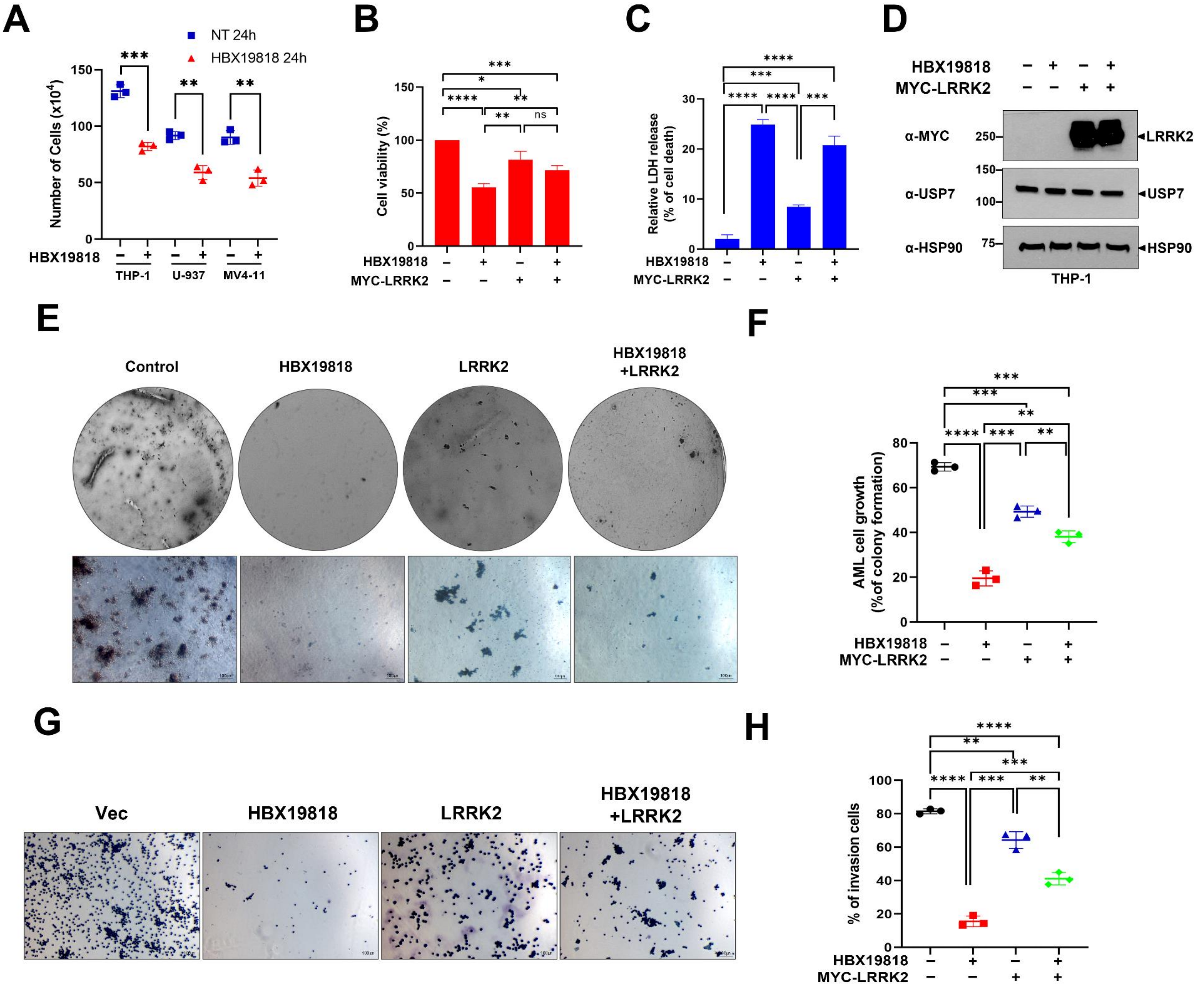

A
NT 24h
HBX19818 24h
Number of Cells (x10^4)
HBX19818
THP-1
U-937
MV4-11
B
Cell viability (%)
HBX19818
MYC-LRRK2
C
Relative LDH release (% of cell death)
D
HBX19818
MYC-LRRK2
α-MYC
α-USP7
α-HSP90
LRRK2
USP7
HSP90
THP-1
E
Control
HBX19818
LRRK2
HBX19818 +LRRK2
F
AML cell growth (%of colony formation)
G
Vec
HBX19818
LRRK2
HBX19818 +LRRK2
H
% of invasion cells

Figure S5. Treatment of AML cells with HBX19818, another selective inhibitor of UPS7, suppresses AML cell growth, and it is partially rescued by LRRK2 overexpression. (A) Where indicated, several AML cell lines, including THP-1, U937, and MV4-11 cells, were treated with 10 µM of HBX19818 for 24 h. Cells were then stained with trypan blue, and the number of live cells was quantified. The results are presented as the mean ± SD from three independent experiments (***$p \leq 0.001$; **$p \leq 0.01$). (B-H) After THP-1 cells were mock-transfected or transfected for 24 h with Myc-LRRK2, cells were left untreated or treated with 10 µM of HBX19818 for additional 24 h. (B) Cell viability was measured after 2 h of incubation with Cell Counting Kit-8 (CCK-8) reagent (****$p \leq 0.0001$; ***$p \leq 0.001$; **$p \leq 0.01$; *$p \leq 0.05$; NS, not-significant). (C) LDH assay was performed to assess cell death (****$p \leq 0.0001$; ***$p \leq 0.001$). (D) Protein expression levels of LRRK2 and USP7 were examined. Cell lysates were subjected to immunoblotting using the indicated antibodies. (E) The results of the colony formation assay are shown. After DNA transfection followed by treatment of HBX19818, cells were then cultured for 21 days in an agarose: media (1:1) mixture. Afterward, colonies were stained with 0.01% crystal violet and observed under a microscope. Scale bars = 100 µm (F) The colony formation ratio relative to the total area was quantified and expressed as a percentage. The results are presented as the mean ± SD from three independent experiments (****$p \leq 0.0001$; ***$p \leq 0.001$; **$p \leq 0.01$). (G) Cell invasion assay results are shown. After 24 h of incubation in the inner chamber of a trans-well, THP-1 cells that invaded into the lower chamber were stained with 0.05% crystal violet and observed under a microscope. Scale bars = 100 µm. (H) The percentage of cells that invaded from the inner chamber of the trans-well to the bottom chamber was quantified. The results are presented as the mean± SD from three independent experiments (****$p \leq 0.0001$; ***$p \leq 0.001$; **$p \leq 0.01$).

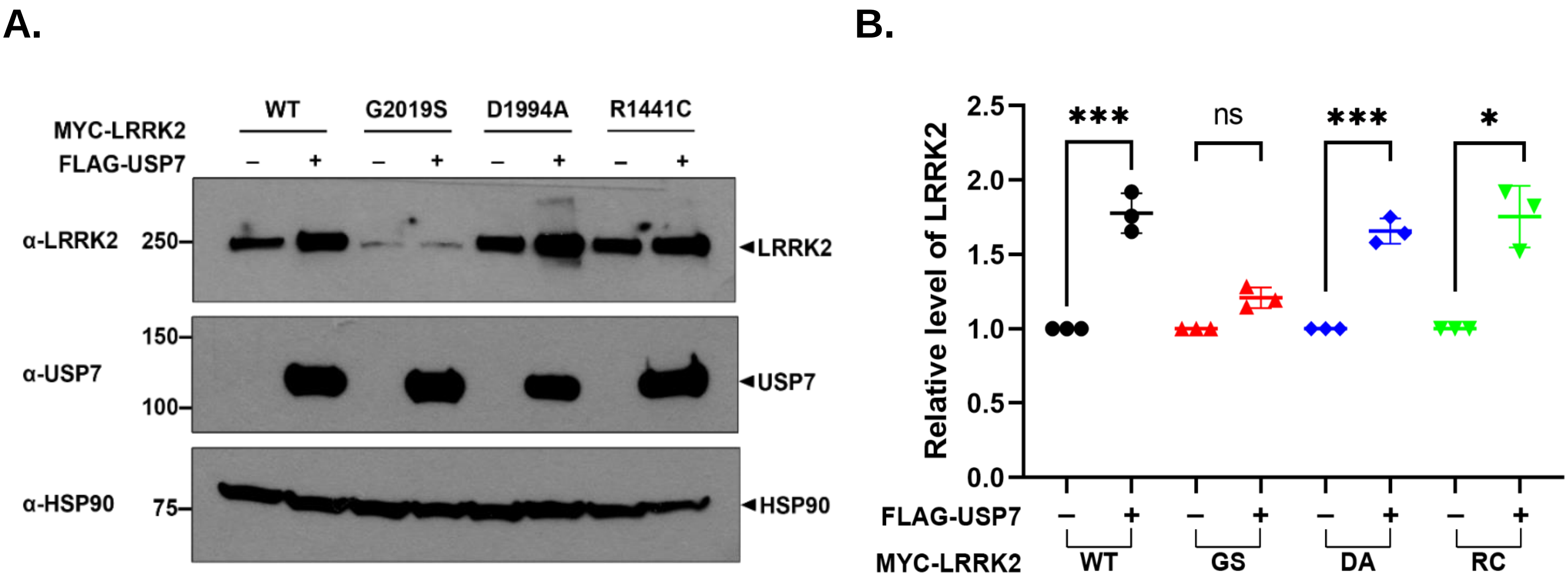

Figure S6. USP7 enhances the levels of wild-type (WT) LRRK2 and two PD-related (D1994 and R1441C) mutants, but not the LRRK2-G2019S mutant (A) Where indicated, HEK293 cells were transfected for 24 h with plasmids encoding Myc-LRRK2-WT, Myc-LRRK2-G2019S, Myc-LRRK2-D1994A, Myc-LRRK2-R1441C, or Flag-USP7-WT, either alone or in combination. Following DNA transfection, cell lysates were subjected to immunoblotting using the indicated antibodies. (B) The relative levels of LRRK2 were quantified, and the results are presented as the mean ± SD from three independent experiments (***$p \leq 0.001$; *$p \leq 0.05$; NS, not-significant). HSP90 served as a loading control.
